# Effect of Photocaged Isopropyl β‐d‐1‐thiogalactopyranoside Solubility on the Light Responsiveness of LacI‐controlled Expression Systems in Different Bacteria

**DOI:** 10.1002/cbic.202000377

**Published:** 2020-10-23

**Authors:** Fabian Hogenkamp, Fabienne Hilgers, Andreas Knapp, Oliver Klaus, Claus Bier, Dennis Binder, Karl‐Erich Jaeger, Thomas Drepper, Jörg Pietruszka

**Affiliations:** ^1^ Institute of Bioorganic Chemistry Heinrich Heine University Düsseldorf at Forschungszentrum Jülich Stetternicher Forst 52426 Jülich Germany; ^2^ Institute of Molecular Enzyme Technology Heinrich Heine University Düsseldorf at Forschungszentrum Jülich Stetternicher Forst 52426 Jülich Germany; ^3^ Institute of Bio- and Geosciences (IBG-1: Biotechnology) Forschungszentrum Jülich Stetternicher Forst 52426 Jülich Germany

**Keywords:** caged compounds, gene expression, optogenetics, photochemistry, synthetic biology

## Abstract

Photolabile protecting groups play a significant role in controlling biological functions and cellular processes in living cells and tissues, as light offers high spatiotemporal control, is non‐invasive as well as easily tuneable. In the recent past, photo‐responsive inducer molecules such as 6‐nitropiperonyl‐caged IPTG (NP‐cIPTG) have been used as optochemical tools for Lac repressor‐controlled microbial expression systems. To further expand the applicability of the versatile optochemical on‐switch, we have investigated whether the modulation of cIPTG water solubility can improve the light responsiveness of appropriate expression systems in bacteria. To this end, we developed two new cIPTG derivatives with different hydrophobicity and demonstrated both an easy applicability for the light‐mediated control of gene expression and a simple transferability of this optochemical toolbox to the biotechnologically relevant bacteria *Pseudomonas putida* and *Bacillus subtilis*. Notably, the more water‐soluble cIPTG derivative proved to be particularly suitable for light‐mediated gene expression in these alternative expression hosts.

## Graphical abstract

**Extended toolbox**: New photocaged IPTG derivatives with various hydrophobicities for light‐induced gene expression were synthesised and tested with three different expression host strains. The most water‐soluble compound showed superior performance, especially in *Bacillus subtilis*.

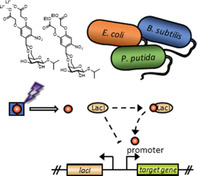

## Introduction

In general, optogenetics combines genetic and optical methods to allow fast control of cellular functions with high spatiotemporal resolution and in a non‐invasive fashion.^[1]^ The control over gene expression by light can basically be realised by employing genetically encoded photoreceptors or chemically photocaged (bio)molecules. Recombinant photoreceptors are typically based on light‐responsive two‐ or one‐component systems, are extensively studied and have been successfully employed as reversible photoswitches for light‐mediated *in vivo* signal transduction in various biological applications.^[2]^

Besides the use of photoreceptors photolabile protecting groups were established as optochemical tools for a variety of diverse applications.^[3]^ In recent years, many approaches were published, in which photocaged compounds have been used for controlling different cellular processes, ranging from cell signalling,[^3b^, ^4^] over drug delivery^[5]^ to gene expression.^[6]^ In this context, especially 2‐nitrobenzyl‐photocaging groups (NB) and their derivatives such as 6‐nitropiperonyl (NP) were commonly used to mediate an adequate and well‐characterised UV‐A light‐triggered release of bioactive molecules.[^3d^, ^7^] To implement caged compounds as versatile optochemical switches, a variety of photolabile protecting groups has been developed focusing on the i) redshifted absorption,[^3b^, ^8^] ii) higher quantum yields^[9]^ and iii) an improved solubility.^[10]^ Especially for *in vivo* approaches an excellent stability towards enzymatic hydrolysis, good biocompatibility, and low overall toxicity of caged compounds (also including the photolysis products) are indispensable.^[11]^ In addition, the extend of the caged compound's solubility could further modulate their ability to pass bacterial cell membranes either through passive processes including free diffusion and porin‐based uptake or by active, membrane transporter‐mediated processes.^[12]^

In the recent past, photoresponsive inducer molecules such as caged derivatives of doxycycline,^[13]^ isopropyl β‐d‐thiogalactopyranoside (IPTG)[^6a^, ^6b^] or several other carbohydrates[^6c^, ^6d^] have been used as irreversible optochemical switches for appropriate microbial expression systems. Especially the applicability of 6‐nitropiperonyl photocaged IPTG (NP‐cIPTG, **1**) for bioengineering approaches using *Escherichia coli* ^[14]^ and *Corynebacterium glutamicum* ^[15]^ as production hosts could be demonstrated. However, a further expansion of the applicability in different expression hosts was for instance hindered by the low water‐solubility of NP‐cIPTG (**1**; 0.7 mm), as appropriately high inducer concentrations were not soluble in the cultivation medium.

**Figure 1:**
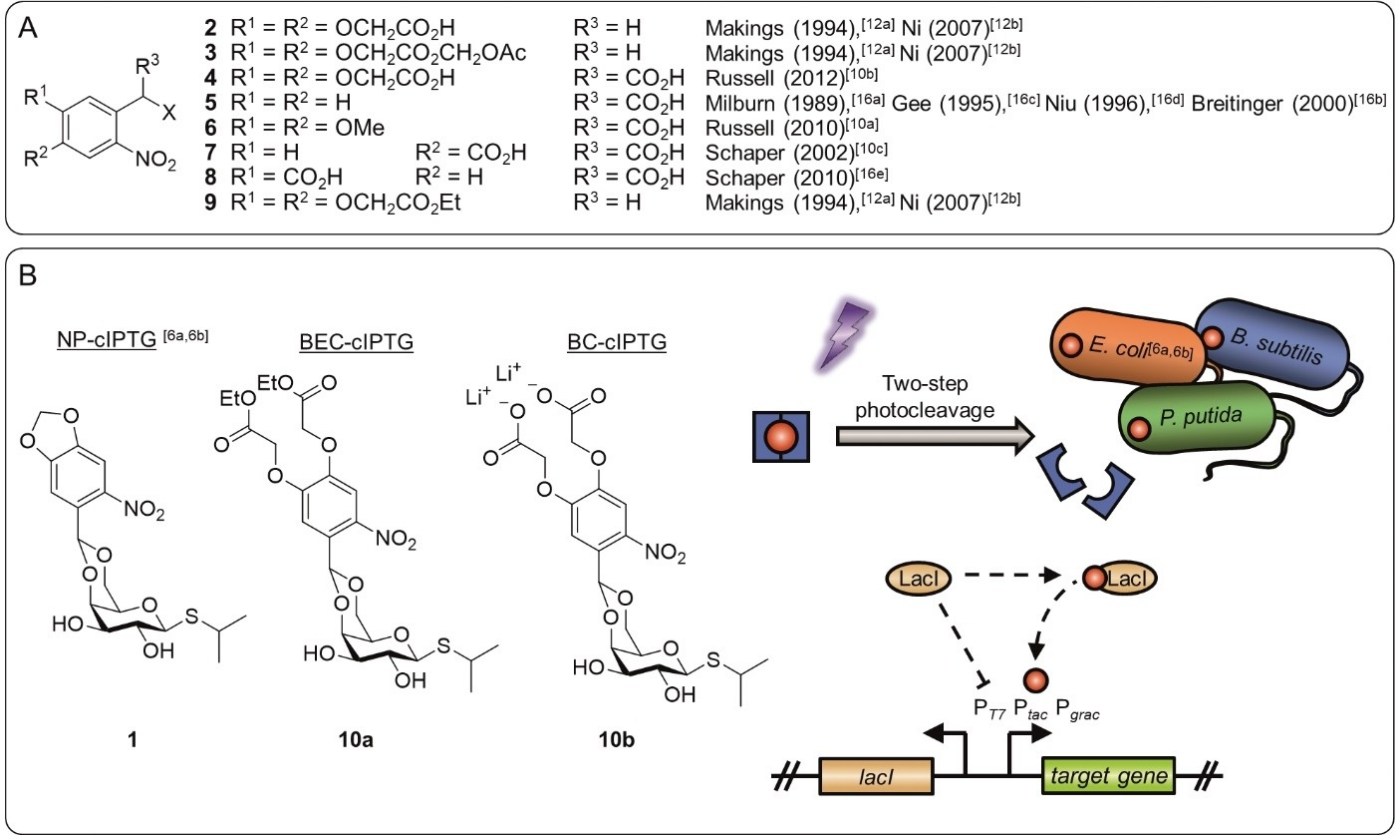
Photolabile protection groups and their application in this work. A) A variety of previously published photolabile protection groups with improved aqueous solubility or membrane permeability based on the NB photocaging group. B) Three photolabile protection groups were used in this work to construct the photocaged IPTG variants NP‐cIPTG (**1**), BEC‐cIPTG (**10 a**) and BC‐cIPTG (**10 b**), strongly differing in their water solubility. These caged inducer molecules (red dot with blue frame) are biologically inactive; however, upon illumination with UV‐A light, their activity can be restored by a two‐step cleavage process. Subsequently, the IPTG binds the repressor protein LacI releasing LacI from the P_T7_, P_tac_ or P_grac_ promoter and thus inducing gene expression. This principle was applied to analyse the effect of cIPTG solubility on the inducibility of LacI repressor‐controlled target gene expression in *E. coli*, *P. putida*, and *B. subtilis*.

Derivatives of the 2‐nitrobenzyl group with improved solubility in aqueous media have been applied before (Figure 1 A). Tsien and co‐worker as well as Ni *et al*. conceived a 4,5‐bis(carboxymethoxy)‐2‐nitrobenzyl protecting group (BC, **2**), which they stated to be highly water‐soluble.

However, they masked the carboxylate **2** as acetoxymethyl ester **3** to facilitate diffusion across cell membranes.[^12a^, ^12b^] Russell *et al*. published a similar derivative **4**, but bearing an additional third carboxy group in the benzylic position, for the synthesis of photolabile tyrosine, whereby a solubility of at least 30 mm was reached.^[10b]^ As the formation of a dioxolane is required for the protection of IPTG, previously reported α‐carboxy‐2‐nitrobenzyl (α‐CNB, **5**–**8**) photocages[^10a^, ^10c^, ^16^] were not considered, because the α‐carboxy‐group increases solubility, but concurrently blocks the position where the dioxolane is later formed.

Based on these results the BC protecting group **2** was chosen in this work as a candidate for the synthesis of a charged, highly water‐soluble photocaged IPTG derivative (Figure 1 B) and was further applied to determine the influence of the solubility and the charge on the inducer uptake through the cell membrane and the resulting expression response. In addition, the 4,5‐bis(ethoxycarbonylmethoxy)‐2‐nitrobenzyl protecting group (BEC, **9**) harbouring lipophilic ester moieties, was selected as an alternative caging group, which might facilitate its passive diffusion across cell membranes. Afterwards, enzymatic hydrolysis of the ester moiety could lead to intracellular accumulation.^[12c]^ To comparatively analyse the effect of caged inducer solubility on light dependent control of gene expression in bacteria, the two new cIPTG derivatives BEC‐cIPTG (**10 a**, derived from **9**) and BC‐cIPTG (**10 b**, derived from **2**) were synthesised and the maximum solubility was quantified. The photophysical properties as well as photolysis in aqueous media were characterised. Subsequently, the *in vivo* applicability of the newly synthesised compounds for light‐inducible gene expression was analysed in comparison to the well‐established NP‐cIPTG (**1**) in *E. coli* in a time‐resolved manner. Finally, we investigated whether optochemical control of gene expression can also be implemented in the alternative expression hosts *Pseudomonas putida* and *Bacillus subtilis*, which exhibit individual morphological and physiological properties. Therefore, we used the photocaged IPTG derivatives **1**, **10 a**, and **10 b** together with appropriate LacI repressor‐controlled expression systems and comparatively evaluated their light‐responsiveness.

## Results

### Synthesis and photochemical properties of cIPTGs

**Scheme 1:**
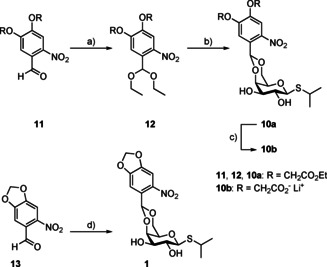
Synthesis of BEC‐, BC‐ and NP‐photocaged IPTGs **10 a**, **10 b** and **1**: a) Triethyl orthoformate, pyridinium *p*‐toluenesulfonate, ethanol, reflux, 19 h (89 %); b) IPTG, *p*‐toluenesulfonic acid, CH_2_Cl_2_, RT, 20 h (45 %); c) 0.2 m LiOH (aq.), MeOH, 0 °C–RT, 1 h (59 %); d) IPTG, sulfuric acid, DMSO, 0 °C–RT, 24 h (21 %).

The BC‐cIPTG (**10 b**) was synthesised in a three‐step reaction (Scheme 1; yield over three steps: 24 %) from 4,5‐bis(ethoxycarbonylmethoxy)‐2‐nitrobenzaldehyde (**11**), which was obtained following the previously reported procedure by Ni *et al*. (see the Supporting Information).^[12b]^ The 2‐nitrobenzaldehyde derivative **11** was reacted with triethyl orthoformate to form the corresponding acetal **12** in 89 % yield, which then was converted to BEC‐cIPTG (**10 a**; 45 %) in a transacetalisation, as the direct acetalization was not feasible. In this step the triethyl orthoformate was preferred to the trimethyl orthoformate due to the occurrence of transesterification during the acid‐catalysed reaction, which was leading towards a mixture of products. After deprotection under basic conditions the BC‐cIPTG (**10 b**) could be obtained in 59 % yield as the corresponding lithium‐salt, which promised advantageous solubility properties compared to the free‐acid. NP‐cIPTG (**1**) was synthesised from 6‐nitropiperonal (**13**) according to literature procedures.[^6a^, ^6b^] The purity of BEC‐cIPTG (**10 a**), BC‐cIPTG (**10 b**) and NP‐cIPTG (**1**) was determined by qNMR (Table S3 in the Supporting Information).

**Scheme 2:**
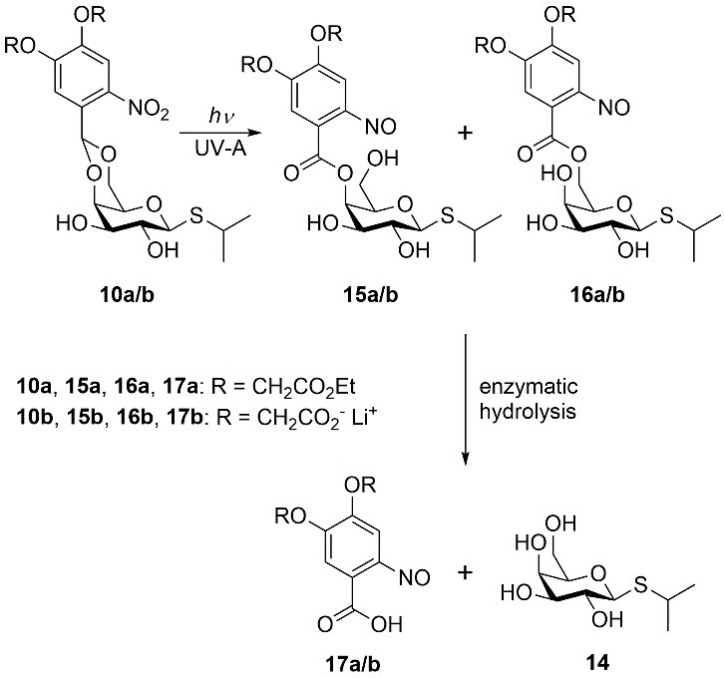
Two‐step release sequence after photolysis of BEC‐ and BC‐photocaged IPTG **10 a** and **10 b** by irradiation with UV‐A light and a subsequent enzymatic hydrolysis by a microbial esterase, as previously described.[^6a^, ^6b^]

Due to the structural similarity of the newly synthesised caged compounds **10 a** and **10 b** to the NP‐cIPTG (**1**), IPTG (**14**) should be released upon UV‐A light exposure in a two‐step photocleavage reaction as previously described.[^6a^, ^6b^] In the first step the irradiation with UV‐A light leads to the formation of ester intermediates **15** and **16**, which might subsequently be cleaved by a microbial esterase. The corresponding nitroso compounds **17** are formed as the photo by‐product (Scheme 2).

The *in vitro* characterisation (Tables 1 and S2, Figures S1–S3) of the new photocaged compounds **10 a** and **10 b** showed uncaging quantum yields (*Φ*_u_) and molar extinction coefficients (*ϵ*) in the range of previously reported caged compounds.[^6d^, ^17^] The resulting photolytic efficiencies (*ϵΦ*_u_) are all in the same order of magnitude. However, more importantly the uncaging half‐life time of the photolytic cleavage amounts to 2.2 min for BEC‐cIPTG (**10 a**), 3.5 min for BC‐cIPTG (**10 b**), and 3.4 min for NP‐cIPTG (**1**). This underlines the fast formation of the ester intermediates **15** and **16** (Figure S4, Table S2). Full photoconversion of the cIPTG variants (1 mm) by irradiation with UV‐A light (375 nm, 6.4 mW cm^−2^) was achieved in less than 30 min for **10 a** and **1**. For derivative **10 b** about ∼5 % of the starting material remained after irradiation for 30 min (Figure S15).

**Table 1:** 

Compound	*λ*_max_ [nm]	*ϵ* ^[a]^ [m ^−1^ cm^−1^]	*t*_0.5_^[b]^ [min]	s^[c]^ [mm]	*Φ*_u_^[d]^	*ϵΦ*_u_^[a]^ [m ^−1^ cm^−1^]
**1^[e]^**	241 336	1690	3.4	0.7	0.50	845
**10 a^[e]^**	298	1810	2.2	<0.1	0.68	1230
**10 b^[f]^**	242 340	3543	3.5	147	0.46	1630
**14**	204	–	–	1941	–	–

[a] *ϵ*=molar extinction coefficient at *λ*=375 nm. [b] *t*_0.5_ = uncaging half‐life time. [c] *s*=solubility in deionised and degassed water. [d] *Φ*_u_=uncaging quantum yield upon 375 nm irradiation. [e] measured in MeOH. [f] measured in sodium phosphate buffer (0.1 mm, pH 7.5).

The BC‐cIPTG (**10 b**) showed a maximum solubility of 147 mm in deionised and degassed water, which is over 200 times higher than the maximum solubility of NP‐cIPTG (**1**),^[6b]^ but only ∼8 % of the maximum solubility of IPTG (**14**) itself (Table 1). Other previously reported photocaged carbohydrates were in the range of 4–58 mm.^[6d]^ In contrast, the BEC‐cIPTG (**10 a**) displayed a more than 7‐times lower solubility of <0.1 mm, as expected due to the ester‐protected carboxylic acids. Since the possible higher membrane permeability of BEC‐cIPTG might result in an improved *in vivo* applicability, this cIPTG derivative was additionally used for further investigations.

### Applicability of cIPTGs for light‐controlled gene expression in bacteria

After the successful synthesis of BEC‐ and BC‐cIPTG (**10 a** and **10 b**), we next analysed whether the different solubility of the cIPTG derivatives (solubility in aqueous solvents: **10 b**≫**1**>**10 a**, see Table 1) affect the inducibility of LacI repressor‐controlled expression systems. The regulatory system, which originally controls the lactose consumption in *E*. *coli*, is one of the most often used regulation mechanisms for triggering heterologous gene expression in this host.^[18]^ The development of different recombinant promoters (e.g., P_*tac*_, P_*trc*_, P_T7_), whose activities can be tightly and gradually controlled by the concentration of the added inducer (e.g., the non‐hydrolysable lactose analogue IPTG) led to its broad applicability in basic research and biotechnological production processes. Furthermore, the development of light‐responsive NP‐cIPTG (**1**) allowed for non‐invasive light‐mediated control of gene expression in *E. coli*.[^6a^, ^6b^, ^6c^] To further optimise light responsiveness of this promising optochemical on‐switch in *E. coli* and to facilitate its transferability to other industrially relevant microbes, we used the following Gram‐negative and ‐positive bacteria as appropriate model hosts offering individual morphological and physiological properties: i) *E. coli* Tuner(DE3) is a lactose permease‐deficient strain and was shown to be well suited for NP‐cIPTG‐based light control of gene expression, because the uptake of appropriate inducers is solely dependent on passive diffusion processes. Previous studies using *E. coli* Tuner(DE3) revealed a very stringently controlled and homogeneous gene expression that gradually responded to changes of illumination time or light intensity.[^6b^, ^6c^, ^14^] ii) *P. putida* KT2440 is a rod‐shaped, Gram‐negative soil bacterium, which offers a pronounced tolerance towards xenobiotics^[19]^ as well as redox stress.^[20]^ Besides its genetic accessibility and its FDA certification as a host‐vector biosafety system,^[21]^ *P. putida* exhibits an extraordinary versatile metabolism that makes it especially suited for a variety of biotechnological applications including the production of various high‐value natural products and their derivatives.^[22]^ iii) *Bacillus subtilis* DB430 is a Gram‐positive bacterium commonly used as a “microbial cell factory” for high‐level production and secretion of proteins for industrial applications.^[23]^ In contrast to the Gram‐negative bacteria used in this study, *B. subtilis* possesses a more rigid and thick cell wall which might act as an additional diffusion barrier for the photocaged IPTG molecules, but lacks an outer membrane. For all the here tested bacterial hosts, expression systems encompassing LacI‐controlled, IPTG‐inducible promoters have been successfully established in recent studies (Table S1).[^6b^, ^18^, ^22c^, ^24^]

To exclude detrimental effects of the new caged inducers or UV‐A light exposure on cell viability, we first analysed the growth of *E. coli*, *P. putida* and *B. subtilis* cells in the presence of the cIPTG derivatives **10 a** and **10 b** as well as their corresponding photoproducts in comparison to conventional IPTG (**14**). For these studies, we used inducer concentrations that were sufficient to fully induce reporter gene expression in the respective expression hosts (Figure S5). Comparative growth of all strains clearly demonstrated that UV‐A light exposure (30 min, 365 nm, ∼1 mW cm^−2^) did not lead to considerable growth impairments in the presence (Figure S6) or absence (Figure S7) of IPTG (**14**) and its photocaged derivatives **1**, **10 a** and **10 b**. Furthermore, the stability of **1**, **10 a** and **10 b** were analysed by measuring the fluorescence intensity of cultures in the dark (Figure S6 A). The data clearly reveals a pronounced *in vivo* stability of the new cIPTG derivatives **10 a** and **10 b** over 20 h in LB medium at 30 °C.

**Figure 2:**
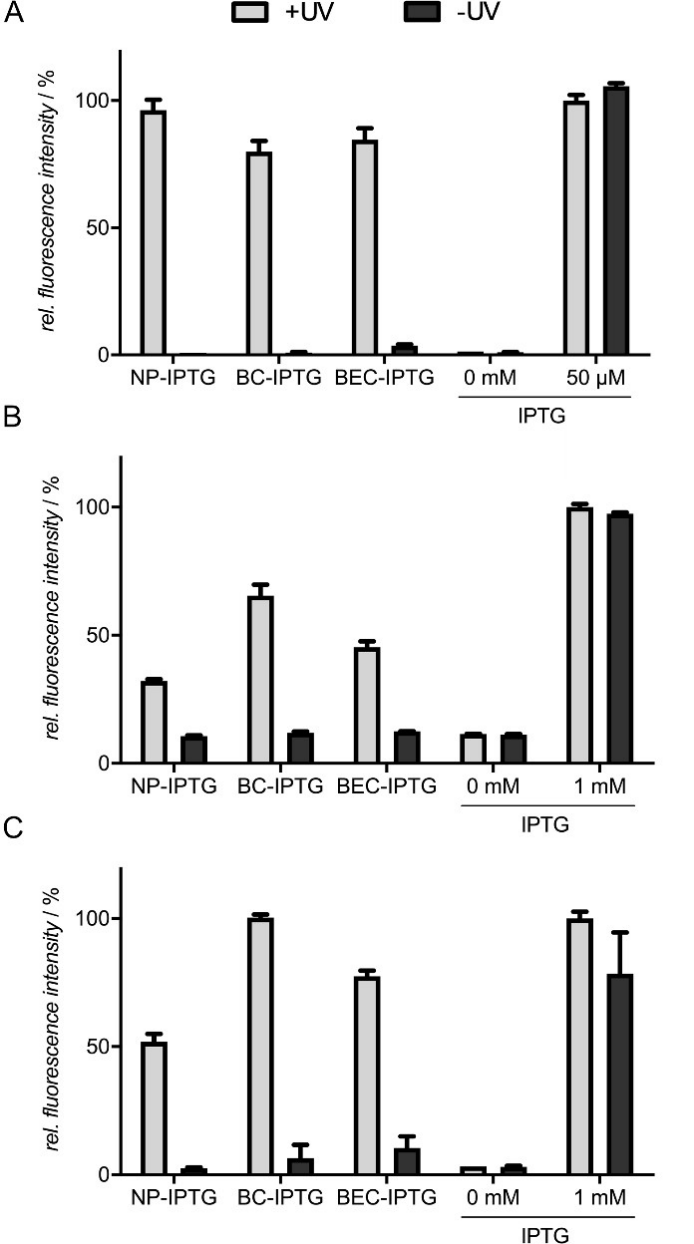
Light‐controlled gene expression in A) *E. coli* Tuner(DE3)/pRhotHi‐2‐lacI‐EYFP, B) *P. putida* KT2440/pVLT33‐GFPmut3 and C) *B. subtilis* DB430/pHT01‐sfGFP using NP‐, BC‐, and BEC‐cIPTG. A) *In vivo* eYFP fluorescence (*λ*_ex_=508 nm, *λ*_em_=532 nm) of *E. coli* cultures supplemented with 50 μm of each cIPTG variant is shown in relation to a 50 μm IPTG (**14**) after 20 h (stationary growth phase). Induction was performed after 2.5 h by UV‐A light exposure at 365 nm (∼1 mW cm^−2^) for 30 min or the addition of 50 μm **14**. B) *In vivo* GFPmut3 fluorescence (*λ*_ex_=508 nm, *λ*_em_=532 nm) of *P. putida* cultures supplemented with 1 mm of each cIPTG variant is shown in relation to a 1 mm IPTG (**14**) control after 20 h (stationary growth phase). Induction was performed after 3 h by UV‐A light exposure at 365 nm (∼1 mW cm^−2^) for 30 min or the addition of 1 mm **14**. C) *In vivo* sfGFP fluorescence (*λ*_ex_=488 nm, *λ*_em_=520 nm) of culture*s* supplemented with 1 mm of each cIPTG variant is shown in relation to a 1 mm IPTG (**14**) control after 20 h. Induction was performed after 5 h by UV‐A light exposure at 365 nm (∼1 mW cm^−2^) for 30 min or the addition of 1 mm **14**. *In vivo* fluorescence intensities were normalized to cell densities, and values are means of triplicate measurements. Error bars indicate the standard deviations.

*Expression studies in E. coli*: To further evaluate the applicability of the new cIPTG derivatives **10 a** and **10 b** in comparison to **1** in *E. coli*, we used the well‐established strain *E. coli* Tuner(DE3) carrying the eYFP expression vector pRhotHi‐2‐lacI‐EYFP.[^6b^, ^14^] Initially, we could observe that, in contrast to the variants **1** and **10 a** which form an emulsion‐like structure at relevant concentrations in LB medium without considerable amounts of ethanol or DMSO, variant **10 b** can be completely dissolved in the cultivation medium, superseding the use of additional solvents. To compare the UV‐A light‐induced gene expression mediated by differently soluble photocaged IPTG variants during *E. coli* cultivation, light exposure was carried out for 30 min in order to ensure sufficient photoconversion of **1**, **10 a** and **10 b** (Figure S4). First, the general applicability of cIPTG variants was evaluated by analysing eYFP expression in cultures that reached the stationary growth phase. As shown in Figure 2A, illumination of the already established NP‐cIPTG resulted in comparable eYFP expression levels as in the control experiment, where conventional IPTG (**14**) was added. In contrast, the new water‐soluble BC‐cIPTG (**10 b**) and the more hydrophobic BEC‐cIPTG (**10 a**) led to a slight decrease of reporter gene expression in this experimental setup.

To analyse the properties of the cIPTG variants in more detail, eYFP expression was subsequently online monitored during batch cultivation of *E. coli*. Illumination of BC‐cIPTG (**10 b**) resulted in the fastest induction response in the early logarithmic growth phase (∼4–7 h after inoculation) as also indicated by a lower half‐maximal responsiveness with *t*_0.5 final_=4.16 h when compared to NP‐cIPTG (**1**) and BEC‐cIPTG (**10 a**; *t*_0.5 final_=4.41 and 4.51 h, respectively, Table S4 and Figure S8). Thus, these results give a first indication that NB caging group derivatives with improved water‐solubility such as BC might slightly facilitate the overall uptake of cIPTG in *E. coli*. However, the lower final eYFP expression levels in the respective cultures point to a less efficient enzymatic release of IPTG from ester intermediates **15** and **16**, which is eventually caused by the increasing size of these photolabile protecting groups. All in all the differential solubility of tested cIPTG variants in aqueous solvents seems to play a minor role for optochemical *in vivo* applications in *E. coli*, since only marginal differences of light‐controlled gene expression could be observed.

*Expression studies in P. putida*: Next, we analysed whether the optochemical cIPTG/LacI system can be transferred to the Gram‐negative bacterium *P. putida* KT2440 and if the solubility of the caged inducer has an effect on its *in vivo* applicability. In the following experiments, we used *P. putida* KT2440 carrying the expression vector pVLT33 harbouring a GFPmut3 gene, which is under control of the P_*tac*_ promoter (Table S1), and the same experimental setup as established for reference strain *E. coli* Tuner(DE3).

Because we could observe only basal induction of gene expression when 50 μm IPTG (**14**) was added to *P. putida* expression cultures (Figure S5), 1 mm of each IPTG derivative was used. As depicted in Figure 2B, the comparison of GFPmut3 fluorescence in *P. putida* cultures that reached the stationary growth phase demonstrates an induction of reporter gene expression of about 70 % for BC‐cIPTG (**10 b**) when compared to conventional IPTG (**14**). In contrast, the use of NP‐ and BEC‐cIPTG (**1** and **10 a**) led to a lower induction response of ∼50 % or less. For BC‐cIPTG (**10 b**) the maximal responsiveness value *t*_0.5 final_ of 2.62 h is significantly slower than IPTG (**14**; *t*_0.5 final_=1.41 h; Figure S8 and Table S4). In summary, cIPTG constitutes an optochemical tool that can be used as an optogenetic switch for LacI‐controlled expression systems in *P. putida*, but comparative expression studies revealed that modified IPTG variants **10 a**, **10 b** and **1** work less efficient than in *E. coli*. Remarkably, only the variant BC‐cIPTG (**10 b**) that offers an increased solubility in aqueous solution showed a satisfactory applicability for controlling gene expression by light. Similar to the *E. coli* Tuner(DE3), *P. putida* lacks a specific lactose permease.^[30]^ Therefore, IPTG can only pass the cytoplasmic membrane *via* passive diffusion processes. Furthermore, in pseudomonads including *P. putida*, the outer membrane exhibits a reduced permeability as compared to *E. coli*. The uptake of small water‐soluble molecules is mainly mediated by a defined set of specific porins such as OprF, which is characterised by a significantly slower diffusion rate compared to the more unspecific *E. coli* porins OmpF and OmpC.[^25^, ^26^] As a consequence, the water‐soluble compound **10 b** could be transported over the outer membrane in a slower process.

*Expression studies in B. subtilis*: The Gram‐positive bacterium *B. subtilis* was used as an expression host to determine the effect of inducer solubility on the uptake process, which is here solely influenced by the permeability of the cytoplasmic membrane and the surrounding cell wall. As this bacterium is not able to use lactose as a carbon source, and a lactose permease‐encoding gene could not be identified in the genome,^[27]^ the uptake of inducer molecules is most probably restricted to passive diffusion. To evaluate the cIPTG applicability, we used the *B. subtilis* DB430/pHT01‐sfGFP strain, where fluorescence reporter expression is driven by the LacI‐controlled P_*grac*_ promoter.^[24b]^ Similar to *P. putida*, we added the respective inducer at a concentration of 1 mm to ensure full induction of recombinant gene expression (Figure S5). Remarkably, illumination of BC‐cIPTG (**10 b**) led to a strong and fast induction response comparable to the results obtained with IPTG (**14**; Figures 2C and S8, Table S4). In contrast, the induction with BEC‐cIPTG (**10 a**) led to a sfGFP expression level of around 75 % in comparison to IPTG (**14**), while addition of NP‐cIPTG (**1**) resulted in only 50 % sfGFP fluorescence. Based on this observation, we cannot exclude that the cell wall of *B. subtilis*, which is much thicker (20–80 nm) than in Gram‐negative organisms (5–10 nm),^[28]^ is less permeable for the more hydrophobic cIPTG variants. In addition, the extremely fast responsiveness of BC‐cIPTG (**10 b**) in *B. subtilis* (*t*_0.5 final_∼2.3 h), which also outperforms the respective induction response in *E. coli* (*t*_0.5 final_∼4.3 h), might indicate an efficient catalytic cleavage of the ester intermediate after photoconversion. It should be noted that addition of BC‐ and BEC‐cIPTG resulted in an increased basal target gene expression in non‐illuminated cultures, which might be due to a slightly reduced stability of these cIPTG derivatives probably caused by a minimal catalytic release of the respective caging groups.

*Analysis of expression heterogeneity*: Finally, we elucidated, if the differential solubility of the applied cIPTG derivatives has an effect on the expression heterogeneity. For *E. coli* strain Tuner(DE3), we have previously proven a homogeneous induction response for both IPTG (**14**) and NP‐cIPTG (**1**), which is primarily due to the absence of the permease and the resulting inducer uptake by diffusion.^[6b]^ In contrast, for *Bacillus* species considerable expression heterogeneities are frequently described.^[29]^ For the direct comparison of expression heterogeneity, fluorescence of the reporter proteins was determined at the single‐cell level in light‐exposed and non‐illuminated cell cultures of *E. coli* and *B. subtilis* using flow cytometry. The results indicate that reporter gene expression was induced homogenously in *E. coli* cells irrespective of the added cIPTG variant (Figure S9 A) thereby corroborating observations from microfluidic investigations with NP‐cIPTG (**1**).^[6b]^ Similarly, the differential solubility of cIPTG variants did not affect the rate of expression heterogeneity in *B. subtilis* although it is generally more pronounced than in *E. coli* (Figure S9 B). Thus, expression heterogeneity is not provoked by a varying efficiency of inducer uptake.

## Discussion

We developed the two new cIPTG derivatives **10 a** and **10 b** with varying hydrophobicity and aimed to analyse whether the change of cIPTG solubility affects the inducibility of LacI repressor‐controlled target gene expression in *E. coli, P. putida* and *B. subtilis*. In the here presented *in vivo* studies, the derivatives are stable against spontaneous hydrolysis and did not induce elevated basal expression of target genes in the dark. In *E. coli*, only marginal differences of light‐controlled gene expression could be observed for the new cIPTG variants in comparison to the well‐established NP‐cIPTG (**1**). Nevertheless, the increased water‐solubility of derivative **10 b** and its homogeneous dispersion without addition of an organic cosolvent, noticeably improves the applicability of this cIPTG derivative. The transfer to *P. putida* and *B. subtilis* clearly demonstrated that the solubility of photocaged inducer molecules is an important aspect that has to be considered for the establishment of a light‐controlled expression system. Here, BC‐cIPTG (**10 b**), the variant that offers an increased solubility in aqueous solution, resulted in high expression levels together with a comparable or even increased induction factor in comparison to IPTG (for direct comparison of cIPTG derivatives’ induction factors see Table S5). In this context it should be noted that, besides the improved solubility in microbial cultivation media, the diverging hydrophobicity of the cIPTG variants as well as the negative charge in case of BC‐IPTG might additionally affect the complex processes that are involved in light‐induced gene expression. These processes include i) the efficiency of photoconversion under the applied cultivation and illumination conditions, ii) the enzymatic hydrolysis of cIPTG ester intermediates by cytoplasmic, periplasmic or extracellular esterases, and iii) the individual permeability of cell membranes for cIPTG, the ester intermediates or released inducer. Thus, the individual physiological and morphological properties of the chosen microbial expression host might exhibit relevant differences such as the respective membrane composition or the ability for active inducer uptake *via* appropriate transporters. In Gram‐negative bacteria, for example, the inducer has to pass two membranes, a process that occurs through i) free diffusion (both membranes), ii) passive transport processes involving unspecific or specific porins (outer membrane), and iii) active transport mechanisms that are facilitated by suitable permeases (cytoplasmic membrane). In Gram‐positive bacteria, even though only one membrane needs to be passed, the surrounding cell wall is much thicker than in Gram‐negative hosts and thus a distinct interaction with the differently soluble cIPTG variants might additionally influence their uptake. However, to unravel the role of individual properties of respective bacterial strains for cIPTG uptake and IPTG release, further experiments have to be performed in future studies.

In conclusion, we have constructed two new caged IPTG variants, characterised their (photo)chemical properties and demonstrated an easy applicability for the light‐mediated control of gene expression in Gram‐negative and Gram‐positive bacteria. Because of their differential solubility, BC‐, NP‐ and BEC‐cIPTG constitute a valuable “starter set” which enables an easy access to a robust, light‐responsive expression system in a broad variety of different hosts. Due to the non‐invasive nature, the here presented optochemical on‐switches additionally allow the external triggering of gene expression in closed biological systems thereby making, for example, anaerobic expression hosts more accessible in the near future.

## Experimental Section

**General remarks**: All chemicals for synthesis were obtained from commercial suppliers and used without further purification unless stated otherwise. Solvents were reagent grade and were dried as well as purified by common methods. Thin‐layer chromatography (TLC) was performed using pre‐coated silica gel plates (Polygram® SIL G/UV, Macherey‐Nagel) and components were visualised by oxidative staining or UV light. Flash chromatography was performed on silica gel (Merck silica gel 60 (0.063–0.200 μm) and solvents for flash chromatography (petroleum ether/ethyl acetate) were distilled prior to use. Optical rotation was determined at 20 °C on a Perkin Elmer Polarimeter 241 MC against sodium D‐line and melting points were recorded using a Büchi melting point B‐545 apparatus. The NMR spectra (^1^H and ^13^C) were measured at 20 °C on a Bruker Avance/DRX 600 spectrometer in deuterated solvents (CDCl_3_, [D_6_]DMSO, D_2_O). The chemical shifts are given in ppm relative to the solvent (^1^H: CDCl_3_=7.26 ppm, ^1^H: [D_6_]DMSO=3.31 ppm or ^1^H: D_2_O=4.79 ppm/^13^C: CDCl_3_=77.16 ppm or ^13^C: [D_6_]DMSO=39.52 ppm). Signals were assigned by means of H COSY, HSQC and HMBC experiments. The IR spectra were recorded with a Perkin Elmer SpectrumOne IR‐spectrometer ATR (Waltham, USA). HRMS (ESI) spectra were recorded by the centrum of analytics of the Heinrich Heine University. UV/Vis absorption spectra were recorded on a Genesys 10S UV/VIS Spectrophotometer (Thermo Scientific) and uncaging experiments were performed in a quartz cuvette with the LUMOS 43® from Atlas Photonics at 375 nm. Light intensity was quantified using a Thermal Power Sensor (S302 C, Thorlabs Inc, USA) and the decay was detected by a Jasco HPLC system [column: Hyperclone 5 μ ODS (C18) 120 (Phenomenex)] combined with an UV/Vis‐detector.

**Synthesis of 4,5‐Bis(ethoxycarbonylmethoxy)‐2‐nitrobenzylaldehyde diethyl acetal (12)**: To a solution of 4,5‐bis(ethoxycarbonylmethoxy)‐2‐nitrobenzaldehyde (**11**) (3.00 g, 8.44 mmol) in ethanol (50 mL) triethyl orthoformate (1.88 g, 12.6 mmol, 1.50 equiv.) and pyridinium *p*‐toluenesulfonate (424 mg, 1.69 mmol, 0.20 equiv.) were added and heated under reflux for 19 h. A dean‐stark trap filled with molecular sieve (3 Å) was utilised for the constant removal of water. After the reaction was completed as indicated by TLC, it was washed with saturated NaHCO_3_ solution. The aqueous phase was then extracted with CH_2_Cl_2_ and the combined organic phase was dried with anhydrous Na_2_SO_4_ and concentrated under reduced pressure. The residue was purified by flash column chromatography on SiO_2_ (petroleum ether/ethyl acetate 85 : 15) to yield a yellow solid (3.22 g, 7.51 mmol, 89 %). *R*_f_=0.25 (petroleum ether/ethyl acetate 80 : 20) m.p. 62.1 °C; ^1^H NMR (600 MHz, [D_6_]DMSO): *δ*=1.12 (t, ^3^ *J*_2′,1′_=7.1 Hz, 6 H, 2′‐*H*), 1.22 (t, ^3^ *J*_11,10 and 11′,10′_=7.1 Hz, 6 H, 11‐*H* and 11′‐*H*), 3.50 (dq, ^2^ *J*_1′a,1′b_=9.3 Hz, ^3^ *J*_1′a,2′_=7.1 Hz, 2 H, 1′_a_‐*H*), 3.62 (dq, ^2^ *J*_1′b,1′a_=9.3 Hz, ^3^ *J*_1′b,2′_=7.1 Hz, 2 H, 1′_b_‐*H*), 4.18 (q, ^3^ *J*_10,11 or 10′,11′_=7.1 Hz, 2 H, 10‐*H* or 10′‐*H*), 4.19 (q, ^3^ *J*_10,11 or 10′,11′_=7.1 Hz, 2 H, 10‐*H* or 10′‐*H*), 4.96 (s, 2 H, 8′‐*H*), 4.99 (s, 2 H, 8‐*H*), 5.88 (s, 1 H, 7‐*H*), 7.09 (s, 1 H, 6‐*H*), 7.57 ppm (s, 1 H, 3‐*H*); ^13^C NMR (151 MHz, [D_6_]DMSO): *δ*=14.0 (*C*‐11 and *C*‐11′), 14.9 (*C*‐2′), 60.8 (*C*‐10 or *C*‐10′), 60.9 (*C*‐10 or *C*‐10′), 65.5 (*C*‐8 or *C*‐8′), 65.6 (*C*‐8 or *C*‐8′), 97.7 (*C*‐7), 110.6 (*C*‐3), 111.5 (*C*‐6), 127.9 (*C*‐1), 141.4 (*C*‐2), 146.5 (*C*‐4), 150.2 (*C*‐5), 168.1 (*C*‐9 or *C*‐9′), 168.1 ppm (*C*‐9 or *C*‐9′); IR (ATR‐film): *ṽ*=2981, 1755, 1692, 1581, 1526, 1446, 1346, 1291, 1196, 1176, 1080, 878, 796 cm^−1^; HRMS (ESI): *m/z* calcd for C_19_H_27_NO_10_^+^: 447.1973 [*M*+NH_4_]^+^; found: 447.1972.

**Synthesis of BEC‐cIPTG (10 a)**: To a solution of 4,5‐bis(ethoxycarbonylmethoxy)‐2‐nitrobenzylaldehyde diethyl acetal (**12**) (1.00 g, 2.33 mmol, 1.50 equiv.) in dry CH_2_Cl_2_ (6 mL) IPTG (370 mg, 1.55 mmol) was added. After 5 min p‐TSA (11.8 mg, 0.06 mmol, 4 mol %) was added to the suspension and it was stirred at room temperature for 20 h. After the reaction was completed as indicated by TLC, a small amount of triethylamine was added and the reaction was concentrated under reduced pressure. The residue was purified by flash column chromatography on SiO_2_ (petroleum ether/ethyl acetate 50 : 50 to 20 : 80) to yield a white solid (403 mg, 0.70 mmol, 45 %). *R*_f_=0.35 (petroleum ether/ethyl acetate 20 : 80); m.p. 104.5 °C; [*α*]= −68 (*c*=1.0 in CHCl_3_); ^1^H NMR (600 MHz, CDCl_3_): *δ*=1.31 (t, ^3^ *J*_11,10 or 11′,10′_=7.2 Hz, 6 H, 11‐*H* and 11′‐*H*), 1.35 (d, ^3^ *J*_CH3‐a/b,SCH_=6.8 Hz, 3 H, C*H*_3_‐a or C*H*_3_‐b), 1.36 (d, ^3^ *J*_CH3‐a/b,SCH_=6.8 Hz, 3 H, C*H*_3_‐a or C*H*_3_‐b), 2.56 (brs, 2 H, 2′′‐O*H* and 3′′‐O*H*), 3.25 (septet, ^3^ *J*_SCH,CH3‐a/b_=6.8 Hz, 1 H, SC*H*), 3.52 (dt, ^3^ *J*_5′′,6′′_ =1.7 Hz, ^3^ *J*_5′′,4′′_=1.2 Hz, 1 H, 5′′‐*H*), 3.64–3.70 (m, 2 H, 2′′‐*H* and 3′′‐*H*), 4.08 (dd, ^2^ *J*_6′′b,6′′a_=12.5 Hz, ^3^ *J*_6′′b,5′′_=1.7 Hz, 1 H, 6′′‐*H*_b_), 4.24–4.31 (m, 6 H, 10‐*H /* 10′‐*H* / 4′′‐*H* / 6′′‐*H*_a_), 4.41 (d, ^3^ *J*_1′′,2′′_=8.7 Hz, 1 H, 1′′‐*H*), 4.77 (s, 2 H, 8‐*H*), 4.82 (s, 2 H, 8′‐*H*), 6.21 (s, 1 H, 7‐*H*), 7.35 (s, 1 H, 6‐*H*), 7.54 ppm (s, 1 H, 3‐*H*); ^13^C NMR (151 MHz, CDCl_3_): *δ*=14.3 (*C*‐11 or *C*‐11′), 14.3 (*C*‐11 or *C*‐11′), 24.1 (*C*H_3_‐a or *C*H_3_‐b), 24.3 (*C*H_3_‐a or *C*H_3_‐b), 35.5 (S*C*H), 61.8 (*C*‐10 or *C*‐10′), 61.9 (*C*‐10 or *C*‐10′), 66.4 (*C*‐8 or *C*‐8′), 66.6 (*C*‐8 or *C*‐8′), 69.8 (*C*‐6′′), 70.1 (*C*‐5′′), 70.3 (*C*‐3′′), 73.9 (*C*‐2′′), 76.2 (*C*‐4′′), 85.7 (*C*‐1′′), 96.6 (*C*‐7), 111.5 (*C*‐3), 112.8 (*C*‐6), 127.7 (*C*‐1), 141.3 (*C*‐2), 147.6 (*C*‐4), 151.7 (*C*‐5), 167.9 (*C*‐9 or *C*‐9′), 167.9 ppm (*C*‐9 or *C*‐9′); IR (ATR‐film): *ṽ*=3478, 2967, 2916, 2866, 1747, 1520, 1287, 1176, 1097, 1077, 1027, 989 cm^−1^; UV/Vis (MeOH): *λ*_max_ (*ϵ*)=298 nm (8006 dm^3^ mol^−1^ cm^−1^); HRMS (ESI): *m/z* calcd for C_24_H_37_N_2_O_13_S: 593.2011 [*M*+NH_4_]^+^; found: 593.2011.

**Synthesis of BC‐cIPTG (10 b)**: A solution of BEC‐cIPTG (**10a**) (200 mg, 0.35 mmol) in MeOH (3.5 mL) was cooled to 0 °C and a 0.2 m solution of LiOH (3.5 mL) was added. The reaction mixture was stirred for 1 h at room temperature. After the reaction was completed as indicated by TLC, the MeOH was evaporated under reduced pressure and the remaining solution was lyophilised overnight. The residue was suspended in THF, sonicated for 15 min and filtrated. After washing with small amounts of cold THF a white solid (107 mg, 0.21 mmol, 59 %) was obtained. m.p. 190 °C (decay); [*α*]=−92 (*c*=1.0 in H_2_O); ^1^H NMR (600 MHz, D_2_O): *δ*=1.29 (d, ^3^ *J*_CH3‐a,SCH_=6.8 Hz, 3 H, C*H*_3_‐a), 1.31 (d, ^3^ *J*_CH3‐b,SCH_=6.8 Hz, 3 H, C*H*_3_‐b), 3.26 (septet, ^3^ *J*_SCH,CH3‐a/b_=6.8 Hz, 1 H, SC*H*), 3.66 (t, ^3^ *J*_5′′,6′′_=9.8 Hz, 1 H, 2′′‐*H*), 3.71–3.82 (m, 2 H, 3′′‐*H*, 5′′‐*H*), 4.18 (m, 2 H, 6′′‐*H*), 4.37 (d, ^3^ *J*_4′′,3′′_=3.6 Hz, 1 H, 4′′‐*H*), 4.60 (s, 2 H, 8′‐*H*), 4.62 (d, ^3^ *J*_1′′,2′′_=9.8 Hz, 1 H, 1′′‐*H*), 4.67 (d, *J*=2.6 Hz, 2 H, 8‐*H*), 6.20 (s, 1 H, 7‐*H*), 7.32 (s, 1 H, 6‐*H*), 7.55 ppm (s, 1 H, 3‐*H*); ^13^C NMR (151 MHz, D_2_O): *δ*=22.9 (CH_3_‐a), 23.3 (CH_3_‐b), 35.0 (SCH), 67.3(C‐8′), 67.4 (C‐8), 69.1 (C‐2′′), 69.3 (C‐6′′), 69.6 (C‐5′′), 72.8 (C‐3′′), 76.5 (C‐4′′), 84.8 (C‐1′′), 96.4 (C‐7), 109.2 (C‐3), 110.8 (C‐6), 126.3 (C‐1), 140.0 (C‐2), 147.2 (C‐4), 151.5 (C‐5), 175.1 (C‐9), 175.4 ppm (C‐9′); IR (ATR‐film): *ṽ*=3124, 3043, 1605, 1522, 1398, 1335, 1277, 1077, 1047, 1024, 824 cm^−1^; UV/Vis (H_2_O): *λ*_max_ (*ϵ*)=245 (5008), 342 nm (3191 dm^3^ mol^‐1^ cm^−1^); HRMS (ESI): *m/z* calcd for C_20_H_29_N_2_O_13_S^+^: 537.1385 [*M*+NH_4_]^+^; found: 537.1382.

**Determination of purity by qNMR**: The purity of the photocaged IPTG derivatives **10 a**, **10 b** and **1** was determined via quantitative NMR. 3,5‐bis(trifluoromethyl)bromobenzene was utilised as internal standard for **10 a** as well as **1** and (methanesulfonyl)methane for **10 b**. The spectra were measured at 20 °C on a Bruker Avance/DRX 600 spectrometer with 64 scans each and 30 μs relaxation time between each scan. The results in Table S3 are means of triplicate measurements.

**Solubility analysis**: The solubility of **10 a**, **10 b** and **14** was determined photometrically at 25 °C using a spectrophotometer Shimadzu UV‐1800 (CPS‐240A). The absorbance of a serial dilution in degassed and deionised water was measured at the absorption maximum of the respective compound. A saturated solution was measured under the same conditions. The solubility was calculated using the Beer‐Lambert law.^[13b]^

**Hydrolytic stability**: For the determination of the hydrolytic stability, a 1 mm solution of the respective compound in methanol or sodium phosphate buffer (0.1 m, pH 7.5) was stored in the dark at room temperature. Samples were removed after 0 and 24 h and analysed by reversed‐phase HPLC.

**Quantification of uncaging half‐life times**: A 1 mm solution of each photocaged compound in methanol or sodium phosphate buffer (0.1 m, pH 7.5) was prepared. In a cuvette 1 mL of this solution was irradiated at room temperature using the LUMOS 43 (375 nm) for a certain time period. The sample was then analysed by reverse phase HPLC Jasco HPLC system [column: Hyperclone 5 μ ODS (C_18_) 120 (Phenomenex)]. For each photocaged compound, the procedure was repeated for different irradiation times. The decrease of concentration was measured by an UV detector.^[6d]^

**Determination of uncaging quantum yields**: The quantum yields of **1**, **10 a** and **10 b** were determined by a relative method in comparison to the quantum yield of 2‐nitropiperonylacetate (NPA‐Ac), as this substrate shows a sufficient similarity to **1**, **10 a** and **10 b**. The procedure was followed as previously described in literature (Figure S4 and Table S2).[^6c^, ^30^]

**Bacterial strains and plasmids**: The *E. coli* strain DH5α^[31]^ was used for all cloning procedures, while the *E. coli* strain S17‐1^[32]^ and Tuner(DE3) (Novagen) were applied for conjugation and expression studies, respectively. All *E. coli* strains, the *P. putida* strain KT2440^[33]^ and the *B. subtilis* strain DB430^[34]^ were grown on LB agar plates or in liquid LB medium (Luria/Miller, Carl Roth®), at 37 °C (*E. coli*) or 30 °C (*P. putida, B. subtilis*). Media were supplemented either with kanamycin (50 μg mL^−1^), gentamicin (25 μg mL^−1^), irgasan (25 μg mL^−1^) or chloramphenicol (5 μg mL^−1^), when appropriate.

All bacterial strains and plasmids used in this study are listed in Table S1, Supporting Information.

**Plasmid construction**: All recombinant DNA techniques were carried out as described by Sambrook *et al*.^[35]^ For the construction of the *B. subtilis* expression vector pHT01‐sfGFP, the sfGFP‐encoding gene was synthesised with flanking NdeI and HindIII restriction sites (Eurofins Genomics, Germany) and subsequently cloned into pET‐22(b) (Novagen, Merck). The resulting vector pET‐22(b)‐sfGFP was used as template for SLIC cloning^[36]^ of a DNA fragment encompassing the *sfgfp* gene into the *B. subtilis* expression vector pHT01 (MoBiTec, Germany) using oligos 3–6 (Table S1, Supporting Information). The *P. putida* expression vector pVLT33‐GFPmut3 was constructed by restriction and ligation. To this end, the *gfpmut3* gene was amplified with flanking *EcoR*I and *Xba*I restriction sites *via* PCR using oligos 1–2 (Table S1). Afterwards, the *EcoR*I/*Xba*I hydrolysed fragment was ligated into the likewise hydrolysed vector backbone pVLT33, resulting in the final expression vector pVLT33‐GFPmut3. Correct nucleotide sequences of all constructs were confirmed by Sanger sequencing (Eurofins Genomics).

**Cultivation conditions**: All *E. coli*, *P. putida* and *B. subtilis* expression cultures were grown in 48‐well Flowerplates® in a BioLector microbioreactor system (m2p labs, Germany) (800 μL LB medium, 1200 rpm, 30 °C), inoculated with an optical density at 580 nm of 0.05. During cultivation, the cell density was measured online through the scattered light intensity at 620 nm. In addition, fluorescence of eYFP and GFP variants (GFPmut3 and sfGFP) were continuously determined using a 508/532 nm and 488/520 nm filter, respectively. cIPTG variants **10 a**, **10 b** or NP‐cIPTG (**1**) were added prior inoculation (final concentration: 50 μm for *E. coli*, 1 mm for *P. putida* and *B. subtilis*; purities of cIPTG variant after synthesis were taken into account accordingly) and expression of reporter genes was induced during the early logarithmic growth phase (after approx. 2.5 h for *E. coli*, 3 h for *P. putida* and 5 h for *B. subtilis*) *via* UV‐A light exposure (VL‐315.BL lamp, Vilber Lourmat, France; ∼1 mW cm^−2^, 30 min exposure) or by addition of equal amounts of conventional IPTG (**14**) after illumination.

**Determination of expression heterogeneity**: For measurement of the expression heterogeneity, *E. coli* and *B. subtilis* cultures were analysed on the single‐cell level by flow cytometry regarding their fluorescence intensity and distribution. Expression cultures were grown as described above and were subsequently sampled as soon as they reached the late logarithmic growth phase (after 8 h for *E. coli* and after 10 h for *B. subtilis*). For this purpose, 40 μL was taken out of the Flowerplate® cultures and added to 600 μL PBS buffer (pH 7.4). Subsequently, the cells were harvested by centrifugation (2 min, 15 000 rpm – 21130×g, RT), adjusted to an optical density of 0.5 (OD_580_) in 100 μL PBS buffer and then transferred into a 96‐well microtiter plate (Greiner Bio‐One GmbH, Frickenhausen, Germany). Finally, these samples were analysed with a flow cytometer (Amnis® CellStreamTM System, Luminex Corporation, Austin, USA). The individual cellular fluorescence brightness was measured using a 488‐nm laser (15 % intensity for *E. coli* and 5 % for *B. subtilis*) for excitation and a 528/46 nm bandpass filter for detection. To exclude cell debris and cell aggregates, the cells were also analysed regarding their size (forward scatter, FSC) and granularity (side scatter, SSC). FSC was measured using an FSC laser (nm) with 80 % of the laser power for *E. coli* and 50 % for *B. subtilis* and a 456/51 nm bandpass filter for detection. For determination of SSC a nm‐light laser with 80 % of the laser power for *E. coli* and 50 % for *B. subtilis* (773/56 nm bandpass filter) was used. Based on the scatter plots, bacterial cells were gated from irrelevant counts for fluorescence analysis. Flow cytometric data were evaluated with the CellStream^TM^ Analysis Software (Merck, now Luminex Corporation).

## Conflict of interest

The authors declare no conflict of interest.

## Supporting information

As a service to our authors and readers, this journal provides supporting information supplied by the authors. Such materials are peer reviewed and may be re‐organized for online delivery, but are not copy‐edited or typeset. Technical support issues arising from supporting information (other than missing files) should be addressed to the authors.

Supplementary

## References

[1] K. Deisseroth , Nat. Methods 2011, 8, 26–29. 21191368 10.1038/nmeth.f.324 PMC6814250

[2a] R. M. Hughes , Crit. Rev. Biochem. Mol. Biol. 2018, 53, 453–474; 30040498 10.1080/10409238.2018.1487382

[2b] Z. Liu , J. Zhang , J. Jin , Z. Geng , Q. Qi , Q. Liang , Front. Microbiol. 2018, 9, 2692; 30467500 10.3389/fmicb.2018.02692 PMC6236058

[2c] E. M. Zhao , Y. Zhang , J. Mehl , H. Park , M. A. Lalwani , J. E. Toettcher , J. L. Avalos , Nature 2018, 555, 683–687; 29562237 10.1038/nature26141 PMC5876151

[2d] S. R. Schmidl , F. Ekness , K. Sofjan , K. N. M. Daeffler , K. R. Brink , B. P. Landry , K. P. Gerhardt , N. Dyulgyarov , R. U. Sheth , J. J. Tabor , Nat. Chem. Biol. 2019, 15, 690–698. 31110305 10.1038/s41589-019-0286-6

[3a] C. Brieke , F. Rohrbach , A. Gottschalk , G. Mayer , A. Heckel , Angew. Chem. Int. Ed. 2012, 51, 8446–8476; 10.1002/anie.201202134 22829531

[3b] A. Bardhan , A. Deiters , Curr. Opin. Struct. Biol. 2019, 57, 164–175; 31132552 10.1016/j.sbi.2019.03.028 PMC7026702

[3c] L. Gardner , A. Deiters , Curr. Opin. Chem. Biol. 2012, 16, 292–299; 22633822 10.1016/j.cbpa.2012.04.010 PMC3424386

[3d] A. Deiters , Curr. Opin. Chem. Biol. 2009, 13, 678–686; 19857985 10.1016/j.cbpa.2009.09.026 PMC2787999

[3e] T. Drepper , U. Krauss , S. Meyer zu Berstenhorst , J. Pietruszka , K.-E. Jaeger , Appl. Microbiol. Biotechnol. 2011, 90, 23–40. 21336931 10.1007/s00253-011-3141-6

[4a] J. Liu , J. Hemphill , S. Samanta , M. Tsang , A. Deiters , J. Am. Chem. Soc. 2017, 139, 9100–9103; 28657738 10.1021/jacs.7b02145 PMC6022368

[4b] D. Kolarski , A. Sugiyama , G. Breton , C. Rakers , D. Ono , A. Schulte , F. Tama , K. Itami , W. Szymanski , T. Hirota , B. L. Feringa , J. Am. Chem. Soc. 2019, 141, 15784–15791. 31509406 10.1021/jacs.9b05445 PMC6787957

[5] J. M. Silva , E. Silva , R. L. Reis , J. Control. Release 2019, 298, 154–176. 30742854 10.1016/j.jconrel.2019.02.006

[6a] D. D. Young , A. Deiters , Angew. Chem. Int. Ed. 2007, 46, 4290–4292; 10.1002/anie.200700057 17458846

[6b] D. Binder , A. Grünberger , A. Loeschcke , C. Probst , C. Bier , J. Pietruszka , W. Wiechert , D. Kohlheyer , K.-E. Jaeger , T. Drepper , Integr. Biol. 2014, 6, 755–765; 10.1039/c4ib00027g 24894989

[6c] D. Binder , C. Bier , A. Grünberger , D. Drobietz , J. Hage-Hülsmann , G. Wandrey , J. Büchs , D. Kohlheyer , A. Loeschcke , W. Wiechert , K.-E. Jaeger , J. Pietruszka , T. Drepper , ChemBioChem 2016, 17, 296–299; 26677142 10.1002/cbic.201500609

[6d] C. Bier , D. Binder , D. Drobietz , A. Loeschcke , T. Drepper , K.-E. Jaeger , J. Pietruszka , Synthesis 2017, 49, 42–52;

[6e] P. M. Kusen , G. Wandrey , V. Krewald , M. Holz , S. Meyer zu Berstenhorst , J. Büchs , J. Pietruszka , J. Biotechnol. 2017, 258, 117–125; 28455204 10.1016/j.jbiotec.2017.04.032

[6f] P. M. Kusen , G. Wandrey , C. Probst , A. Grünberger , M. Holz , S. Meyer zu Berstenhorst , D. Kohlheyer , J. Büchs , J. Pietruszka , ACS Chem. Biol. 2016, 11, 2915–2922. 27570879 10.1021/acschembio.6b00462

[7] J. E. T. Corrie in *Dynamic Studies in Biology: Phototriggers, Photoswitches and Caged Biomolecules*, (Eds.: M. Goeldner, R. Givens), Wiley-VCH, Weinheim, **2005**, pp. 1–94.

[8a] A. Y. Vorobev , A. E. Moskalensky , Comput. Struct. Biotechnol. J. 2019, 18, 27–34; 31890141 10.1016/j.csbj.2019.11.007 PMC6920508

[8b] I. Aujard , C. Benbrahim , M. Gouget , O. Ruel , J.-B. Baudin , P. Neveu , L. Jullien , Chem. Eur. J. 2006, 12, 6865–6879. 16763952 10.1002/chem.200501393

[9] A. Specht , M. Goeldner , Angew. Chem. Int. Ed. 2004, 43, 2008–2012; 10.1002/anie.200353247 15065287

[10a] A. G. Russell , M.-E. Ragoussi , R. Ramalho , C. W. Wharton , D. Carteau , D. M. Bassani , J. S. Snaith , J. Org. Chem. 2010, 75, 4648–4651; 20536152 10.1021/jo100783v

[10b] A. G. Russell , M. J. Sadler , H. J. Laidlaw , A. Gutierrez-Loriente , C. W. Wharton , D. Carteau , D. M. Bassani , J. S. Snaith , Photochem. Photobiol. Sci. 2012, 11, 556–563; 22249211 10.1039/c2pp05320a

[10c] K. Schaper , S. Mobarekeh , C. Grewer , Eur. J. Org. Chem. 2002, 2002, 1037–1046;

[10d] M. Noguchi , M. Skwarczynski , H. Prakash , S. Hirota , T. Kimura , Y. Hayashi , Y. Kiso , Bioorg. Med. Chem. 2008, 16, 5389–5397. 18440235 10.1016/j.bmc.2008.04.022

[11] A. P. Pelliccioli , J. Wirz , Photochem. Photobiol. Sci. 2002, 1, 441–458. 12659154 10.1039/b200777k

[12a] L. R. Makings , R. Y. Tsien , J. Biol. Chem. 1994, 269, 6282–6285; 8119976

[12b] J. Ni , D. A. Auston , D. A. Freilich , S. Muralidharan , E. A. Sobie , J. P. Y. Kao , J. Am. Chem. Soc. 2007, 129, 5316–5317; 17425315 10.1021/ja069361q PMC2536572

[12c] K. M. Schelkle , C. Schmid , K. Yserentant , M. Bender , I. Wacker , M. Petzoldt , M. Hamburger , D.-P. Herten , R. Wombacher , R. R. Schröder , U. H. F. Bunz , Angew. Chem. Int. Ed. 2017, 56, 4724–4728; 10.1002/anie.201612112 28328078

[13a] S. B. Cambridge , D. Geissler , S. Keller , B. Cürten , Angew. Chem. Int. Ed. 2006, 45, 2229–2231; 10.1002/anie.200503339 16506298

[13b] S. B. Cambridge , D. Geissler , F. Calegari , K. Anastassiadis , M. T. Hasan , A. F. Stewart , W. B. Huttner , V. Hagen , T. Bonhoeffer , Nat. Methods 2009, 6, 527–531. 19503080 10.1038/nmeth.1340

[14] G. Wandrey , C. Bier , D. Binder , K. Hoffmann , K.-E. Jaeger , J. Pietruszka , T. Drepper , J. Büchs , Microb. Cell Fact. 2016, 15, 1–16. 27107964 10.1186/s12934-016-0461-3 PMC4842301

[15] D. Binder , J. Frohwitter , R. Mahr , C. Bier , A. Grünberger , A. Loeschcke , P. Peters-Wendisch , D. Kohlheyer , J. Pietruszka , J. Frunzke , K.-E. Jaeger , V. F. Wendisch , T. Drepper , Appl. Environ. Microbiol. 2016, 82, 6141–6149. 27520809 10.1128/AEM.01457-16 PMC5068161

[16a] T. Milburn , N. Matsubara , A. P. Billington , J. B. Udgaonkar , J. W. Walker , B. K. Carpenter , W. W. Webb , J. Marque , W. Denk , Biochemistry 1989, 28, 49–55; 2706267 10.1021/bi00427a008

[16b] H.-G. A. Breitinger , R. Wieboldt , D. Ramesh , B. K. Carpenter , G. P. Hess , Biochemistry 2000, 39, 5500–5508; 10820023 10.1021/bi992781q

[16c] K. R. Gee , L. Niu , K. Schaper , G. P. Hess , J. Org. Chem. 1995, 60, 4260–4263;

[16d] L. Niu , K. R. Gee , K. Schaper , G. P. Hess , Biochemistry 1996, 35, 2030–2036; 8639688 10.1021/bi9516485

[16e] K. Schaper , S. A. Madani Mobarekeh , P. Doro , D. Maydt , Photochem. Photobiol. 2010, 86, 1247–1254. 20880228 10.1111/j.1751-1097.2010.00803.x

[17] J. E. T. Corrie , J. Chem. Soc. Perkin Trans. 1 1993, 2161–2166.

[18] K. Terpe , Appl. Microbiol. Biotechnol. 2006, 72, 211–222. 16791589 10.1007/s00253-006-0465-8

[19a] O. Simon , I. Klaiber , A. Huber , J. Pfannstiel , J. Proteomics 2014, 109, 212–227; 25026441 10.1016/j.jprot.2014.07.006

[19b] Ö. Akkaya , D. R. Pérez-Pantoja , B. Calles , P. I. Nikel , V. de Lorenzo , MBio 2018, 9, e01512–18. 30154264 10.1128/mBio.01512-18 PMC6113623

[20a] M. Fernández , S. Conde , J. de la Torre , C. Molina-Santiago , J.-L. Ramos , E. Duque , Antimicrob. Agents Chemother. 2012, 56, 1001–1009; 22143519 10.1128/AAC.05398-11 PMC3264264

[20b] M. Chavarría , P. I. Nikel , D. Pérez-Pantoja , V. de Lorenzo , Environ. Microbiol. 2013, 15, 1772–1785. 23301697 10.1111/1462-2920.12069

[21] L. F. Kampers , R. J. Volkers , V. A. Martins dos Santos , Microb. Biotechnol. 2019, 12, 845–848. 31199068 10.1111/1751-7915.13443 PMC6680625

[22a] P. I. Nikel , V. de Lorenzo , New Biotechnol. 2014, 31, 562–571; 10.1016/j.nbt.2014.02.006 24572656

[22b] T. Tiso , R. Zauter , H. Tulke , B. Leuchtle , W.-J. Li , B. Behrens , A. Wittgens , F. Rosenau , H. Hayen , L. M. Blank , Microb. Cell Fact. 2017, 16, 225; 29241456 10.1186/s12934-017-0838-y PMC5729600

[22c] P. I. Nikel , V. de Lorenzo , Metab. Eng. 2018, 50, 142–155; 29758287 10.1016/j.ymben.2018.05.005

[22d] E. Martínez-García , V. de Lorenzo , Curr. Opin. Biotechnol. 2019, 59, 111–121; 31048223 10.1016/j.copbio.2019.03.012

[22e] A. Loeschcke , S. Thies , Curr. Opin. Biotechnol. 2020, 65, 213–224; 32498036 10.1016/j.copbio.2020.03.007

[22f] M. R. Incha , M. G. Thompson , J. M. Blake-Hedges , Y. Liu , A. N. Pearson , M. Schmidt , J. W. Gin , C. J. Petzold , A. M. Deutschbauer , J. D. Keasling , Metab. Eng. Commun. 2020, 10, e00119. 32280587 10.1016/j.mec.2019.e00119 PMC7136493

[23] J. van Dijl , M. Hecker , Microb. Cell Fact. 2013, 12, 3. 23311580 10.1186/1475-2859-12-3 PMC3564730

[24a] S. C. Troeschel , S. Thies , O. Link , C. I. Real , K. Knops , S. Wilhelm , F. Rosenau , K.-E. Jaeger , J. Biotechnol. 2012, 161, 71–79; 22440389 10.1016/j.jbiotec.2012.02.020

[24b] H. D. Nguyen , T. T. P. Phan , W. Schumann , Curr. Microbiol. 2007, 55, 89–93; 17624574 10.1007/s00284-006-0419-5

[24c] M. M. Bagdasarian , E. Amann , R. Lurz , B. Rückert , M. Bagdasarian , Gene 1983, 26, 273–282. 6323265 10.1016/0378-1119(83)90197-x

[25] H. Löwe , P. Sinner , A. Kremling , K. Pflüger-Grau , Microb. Biotechnol. 2020, 13, 97–106. 29808622 10.1111/1751-7915.13283 PMC6922520

[26] E. Eren , J. Vijayaraghavan , J. Liu , B. R. Cheneke , D. S. Touw , B. W. Lepore , M. Indic , L. Movileanu , B. van den Berg , PLoS Biol. 2012, 10, e1001242. 22272184 10.1371/journal.pbio.1001242 PMC3260308

[27a] O. Krispin , R. Allmansberger , J. Bacteriol. 1998, 180, 2265–2270; 9555917 10.1128/jb.180.8.2265-2270.1998 PMC107161

[27b] M. Steinmetz , in Bacillus subtilis and Other Gram-Positive Bacteria: Biochemistry, Physiology and Molecular Genetics (Eds.: A. L. Sonenshein , J. A. Hoch , R. Losick ), American Society of Microbiology, Washington, 1993, pp. 157–170.

[28] M. R. J. Salton, K. S. Kim, in *Medical Microbiology*, 4th ed. (Ed.: S. Baron), University of Texas Medical Branch at Galveston, Galveston, **1996**, Chapter 2. 21413252

[29a] K. M. Münch , J. Müller , S. Wienecke , S. Bergmann , S. Heyber , R. Biedendieck , R. Münch , D. Jahn , Appl. Environ. Microbiol. 2015, 81, 5976–5986; 26116677 10.1128/AEM.00807-15 PMC4551236

[29b] T. N. Ploss , E. Reilman , C. G. Monteferrante , E. L. Denham , S. Piersma , A. Lingner , J. Vehmaanperä , P. Lorenz , J. M. van Dijl , Microb. Cell Fact. 2016, 15, 57; 27026185 10.1186/s12934-016-0455-1 PMC4812647

[29c] D. B. Kearns , R. Losick , Genes Dev. 2005, 19, 3083–3094; 16357223 10.1101/gad.1373905 PMC1315410

[29d] D. Dubnau , R. Losick , Mol. Microbiol. 2006, 61, 564–572. 16879639 10.1111/j.1365-2958.2006.05249.x

[30a] F. Bley , K. Schaper , H. Görner , Photochem. Photobiol. 2008, 84, 162–171; 18173716 10.1111/j.1751-1097.2007.00215.x

[30b] B. A. M. Bier, *PhD thesis*, Heinrich Heine University Düsseldorf (Germany), **2011**.

[31] D. Hanahan , J. Mol. Biol. 1983, 166, 557–580. 6345791 10.1016/s0022-2836(83)80284-8

[32] R. Simon , U. Priefer , A. Pühler , Bio/Technology 1983, 1, 784–791.

[33] M. Bagdasarian , R. Lurz , B. Rückert , F. C. H. Franklin , M. M. Bagdasarian , J. Frey , K. N. Timmis , Gene 1981, 16, 237–247. 6282695 10.1016/0378-1119(81)90080-9

[34] R. H. Doi , S.-L. Wong , F. Kawamura , Trends Biotechnol. 1986, 4, 232–235.

[35] J. Sambrook , E. F. Fritsch , T. Maniatis , Molecular Cloning: A Laboratory Manual, Cold Spring Harbor Laboratory Press, Cold Spring Harbor, 1989, p. 1546.

[36] J.-Y. Jeong , H.-S. Yim , J.-Y. Ryu , H. S. Lee , J.-H. Lee , D.-S. Seen , S. G. Kang , Appl. Environ. Microbiol. 2012, 78, 5440–5443. 22610439 10.1128/AEM.00844-12 PMC3416421

