# Supplementary material for: Effect of Photocaged Isopropyl β‐d‐1‐thiogalactopyranoside Solubility on the Light Responsiveness of LacI‐controlled Expression Systems in Different Bacteria
Source: Chembiochem. 2020 Oct 23;22(3):539–47. doi: 10.1002/cbic.202000377 (PMC7894499; doi:10.1002/cbic.202000377)
Supplement: Supplementary file 1 — Supplementary [file CBIC-22-539-s001.pdf]

ChemBioChem

Supporting Information

Effect of Photocaged Isopropyl β-D-1-thiogalactopyranoside Solubility on the Light Responsiveness of LacI-controlled Expression Systems in Different Bacteria

Fabian Hogenkamp[+], Fabienne Hilgers[+], Andreas Knapp, Oliver Klaus, Claus Bier, Dennis Binder, Karl-Erich Jaeger, Thomas Drepper,* and Jörg Pietruszka*

Table of Contents

S1 Supporting methods **S1**

S1.1 Synthesis of 3,4-bis(ethoxycarbonylmethoxy)benzaldehyde (18) S1

S1.2 Synthesis of 4,5-bis(ethoxycarbonylmethoxy)-2-nitrobenzaldehyde (11) S2

S1.3 Bacterial strains and plasmids S3

S2 Supporting data **S4**

S2.1 UV-Vis spectra of compounds S4

S2.2 Determination of uncaging half-life times S6

S2.3 Determination of purity by qNMR S8

S2.4 Determination of IPTG concentrations sufficient for the induction of gene expression in *E. coli*, *P. putida* and *B. subtilis* S9

S2.5 Stability and toxicity of novel photocaged IPTG variants S10

S2.6 Effect of UV-A light illumination on cell growth and fluorescence of *E. coli*, *P. putida* and *B. subtilis* expression cultures S11

S2.7 Comparative analysis of light-responsiveness of cIPTG variants in different bacterial expression systems S12

S2.8 Heterogeneity of light-induced reporter gene expression in *E. coli* and *B. subtilis* using different cIPTG variants S14

S2.9 NMR spectra of compounds S16

S2.10 HPLC-Traces S21

S1 Supporting methods

S1.1 Synthesis of 3,4-bis(ethoxycarbonylmethoxy)benzaldehyde (18)

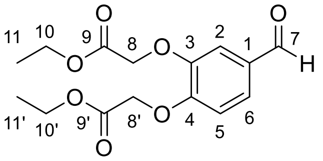

To a solution of 3,4-dihydroxybenzaldehyde (3.00 g, 21.7 mmol) in DMF (42 mL) K_2CO_3 (12.2 g, 86.9 mmol, 4.00 equiv.) was added and it was stirred for 30 min at room temperature. The reaction mixture was cooled to 0 °C and ethyl bromoacetate was added dropwise. The reaction mixture was stirred for additional 30 min at 0 °C, before it was stirred at room temperature for 16 h. The reaction was quenched by addition of water (60 mL). The aqueous phase was extracted with ethyl acetate. The organic phase was washed with saturated NaCl solution, dried with anhydrous $MgSO_4$ and concentrated under reduced pressure. The residue was purified by flash-column chromatography on SiO_2 (petroleum ether/ethyl acetate 7:3) to yield a white solid (5.88 g, 19.0 mmol, 87%). The spectroscopic data are in agreement with previously reported literature values.[1] R_f = 0.27 (petroleum ether/ethyl acetate 7:3); m.p. 55 °C, Lit.[2]: 55 - 56 °C; ^{1}H-NMR (600 MHz, $CDCl_3$): δ = 1.28 (t, $^3J_{11,10 \text{ or } 11',10'}$ = 7.2 Hz, 3 H, 11-*H* or 11′-*H*), 1.29 (t, $^3J_{11,10 \text{ or } 11',10'}$ = 7.2 Hz, 3 H, 11-*H* or 11′-*H*), 4.26 (q, $^3J_{10,11 \text{ or } 10',11'}$ = 7.2 Hz, 4 H, 10-*H* and 10´-*H*), 4.76 (s, 2 H, 8-*H*), 4.80 (s, 2 H, 8´-*H*), 6.92 (d, $^3J_{5,6}$ = 8.3 Hz, 1 H, 5-*H*), 7.37 (d, $^4J_{2,6}$ = 1.9 Hz, 1 H, 2-*H*), 7.47 (dd, $^3J_{6,5}$ = 8.3 Hz, $^4J_{6,2}$ = 1.9 Hz, 1 H, 6-*H*), 9.83 ppm (s, 1 H, 7-*H*); ^{13}C-NMR (151 MHz, $CDCl_3$): δ = 14.27 (*C*-11 and *C*-11′), 61.63 (*C*-10 or *C*-10′), 61.74 (*C*-10 or *C*-10′), 66.25 (*C*-8), 66.28 (*C*-8′), 113.05 (*C*-2), 113.66 (*C*-5), 127.00 (*C*-6), 131.14 (*C*-1), 148.39 (*C*-3), 153.17 (*C*-4), 168.16 (*C*-9′), 168.39 (*C*-9), 190.59 ppm (*C*-7); IR (ATR-film): $\tilde{\nu}$ = 2977, 1755, 1724, 1687, 1585, 1510, 1429, 1207, 1138, 1054, 1024, 671 cm^{-1}; MS (ESI, positive-ion): m/z (%): 333.2 (100) $[M+Na]^+$, 349.1 (40) $[M+K]^+$.

S1.2 Synthesis of 4,5-bis(ethoxycarbonylmethoxy)-2-nitrobenzaldehyde (11)

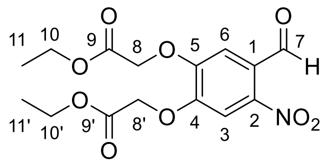

A solution of KNO_3 (2.31 g, 22.8 mmol, 1.25 equiv.) in trifluoroacetic acid (30 mL) was cooled to 0 °C and a solution of 3,4-bis(ethoxycarbonylmethoxy)benzaldehyde (**18**) (5.66 g, 18.2 mmol, 0.9 M) in trifluoroacetic acid was added dropwise. The reaction mixture was stirred for 1 h at 0 °C and for 16 h at room temperature. Then it was concentrated under reduced pressure, the residue was dissolved in ethyl acetate and washed with saturated $NaHCO_3$ and saturated NaCl solution. The organic phase was dried with anhydrous $MgSO_4$ and concentrated under reduced pressure. The residue was purified by flash-column chromatography on SiO_2 (petroleum ether/ethyl acetate 6:4) to yield a yellow solid (4.44 g, 12.5 mmol, 69%). The spectroscopic data are in agreement with previously reported literature values.[1] R_f = 0.44 (toluene/ethyl acetate 85:15); m.p. 94 °C; ^{1}H-NMR (600 MHz, $CDCl_3$): δ = 1.30 (t, $^3J_{11,10\text{ or }11',10'}$ = 7.2 Hz, 3 H, 11-*H* or 11′-*H*), 1.32 (t, $^3J_{11,10\text{ or }11',10'}$ = 7.2 Hz, 3 H, 11-*H* or 11′-*H*), 4.28 (q, $^3J_{10,11\text{ or }10',11'}$ = 7.2 Hz, 2 H, 10-*H* or 10′-*H*), 4.29 (q, $^3J_{10,11\text{ or }10',11'}$ = 7.2 Hz, 2 H, 10-*H* or 10′-*H*), 4.85 (s, 2 H, 8-*H* or 8′-*H*), 4.86 (s, 2 H, 8-*H* or 8′-*H*), 7.34 (s, 1 H, 3-*H*), 7.58 (s, 1 H, 6-*H*), 10.41 ppm (s, 1 H, 7-*H*); ^{13}C-NMR (151 MHz, $CDCl_3$): δ = 14.2 (*C*-11 or *C*-11′), 14.3 (*C*-11 or *C*-11′), 62.0 (*C*-10 or *C*-10′), 62.1 (*C*-10 or *C*-10′), 66.1 (*C*-8 or *C*-8′), 66.4 (*C*-8 or *C*-8′), 110.4 (*C*-6), 112.5 (*C*-3), 126.3 (*C*-1 or *C*-2), 144.2 (*C*-1 or *C*-2), 150.9 (*C*-4 or *C*-5), 151.9 (*C*-4 or *C*-5), 167.3 (*C*-9 or *C*-9′), 167.4 (*C*-9 or *C*-9′), 187.3 ppm (*C*-7); IR (ATR-film): $\tilde{\nu}$ = 2987, 1740, 1687, 1570, 1507, 1283, 1196, 1168, 1070, 1022, 792 cm^{-1}; MS (ESI, positive-ion): m/z (%): 378.2 (50) $[M+Na]^+$.

S1.3 Bacterial strains and plasmids

All bacterial strains, plasmids and oligonucleotides used in this study are listed in Table S1.

Table S1: Bacterial strains, plasmids and oligonucleotides used in this study.

Strains, plasmids, oligonucleotides	Relevant features, description or sequences[a]	References
	**Strains**	
***E. coli* DH5α**	*F⁻ Φ80lacZΔM15 Δ(lacZYA-argF) U169 recA1 endA1 hsdR17 phoA supE44 thi-1 gyrA96 relA1 deoR*	[3]
***E. coli* S17-1**	Ec294::[RP4-2 (Tc^R::Mu)(Km^R::Tn7)] *recA, thi, pro, hsdR⁻ hsdM⁺* Tp^R Sm^R	[4]
***E. coli* Tuner(DE3)**	*F⁻ompT hsdS*$_B$ *(*r_B^- m_B^-*) gal dcm lacY1(DE3)*	Novagen, Merck KGaA
***P. putida* KT2440**	Wild-type	[5]
***B. subtilis* DB430**	*trpC2 his nprE aprE bpf ispI*	[6]
	**Plasmids**	
**pRhotHi-2-lacI-EYFP**	pBBR1-MCS-derivative, Km^R, Cm^R, pBBR22b-*lacI*, P_{T7}-lacO-MCS with *Nde*I *Xho*I inserted *eyfp*	[7]
**pVLT33**	R6K, RSF1010 *lacI*^{q}, Kan^R, P_{tac}	[8]
**pVLT33-GFPmut3**	R6K, RSF1010 *lacI*^{q}, Kan^R, P_{tac} with *EcoR*I *Xba*I inserted *gfpmut3*	This work
**pET-22(b)-sfGFP**	Ap^R, *lacI*, P_{T7}-lacO-MCS with *Nde*I *Hin*dIII inserted *sfgfp*	This work
**pHT01**	Pgrac-lacO-MCS, *lacI*, Cm^R	MoBiTec, Germany
**pHT01-sfGFP**	Pgrac-lacO-MCS, *lacI*, Cm^R, *sfgfp*	This work
	**Oligonucleotides**	
**1) EcoRI_GFP_fw**	Binds at the 5’ end of the *gfpmut3* gene, inserts *EcoR*I site<br>5’-ATATGAATTCATGGTACCAAGTAAAGGAG-3’	This work
**2) XbaI_GFP_rev**	Binds at the 3’ end of the *gfpmut3* gene, inserts *Hind*III site<br>5’-ATATTCTACATTATTTGTATAGTTCATC CATGC-3’	This work
**3) pHT01_fw**	Amplification of pHT01 plasmid for SLIC cloning<br>5’- GAAGGGAATTCATATTACTTAGAGGAT ACT-3′	This work
**4) pHT01_rev**	Amplification of pHT01 plasmid for SLIC cloning<br>5’- CCTCCTTTAATTGGGAATTGTTATCCG-3’	This work
**5) sfgfp_fw**	Binds at the 5’end of the *sfgfp* gene for SLIC cloning<br>5’- GGATAACAATTCCCAATTAAAGGAGGA GATATACATATGAGCAAAGGAGAAGA-3’	This work
**6) sfgfp_rev**	Binds at the 3’ end of the *sfgfp* gene for SLIC cloning<br>5’- GTATCCTCTAAGTAATATGAATTCCCTTC CAGCCGGATCTCAGTGGT-3’	This work

S2 Supporting data

S2.1 UV-Vis spectra of compounds

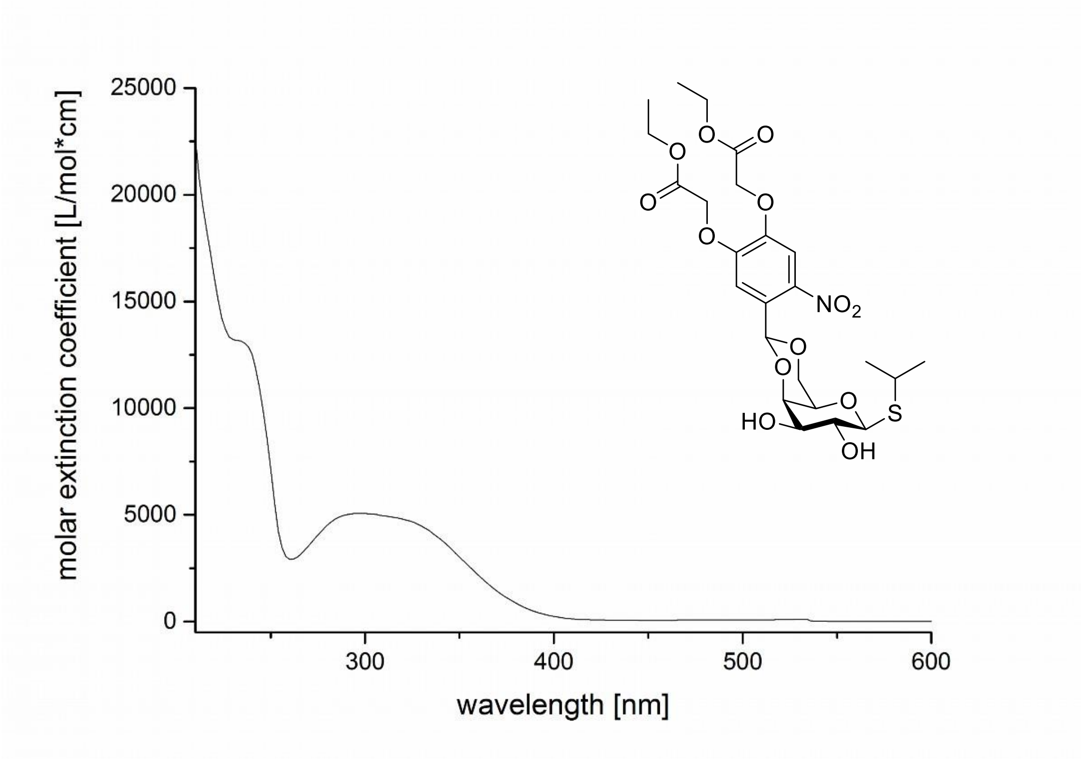

Figure S1: UV-Vis spectrum of compound **10a** (0.125 mM in MeOH, 25 °C).

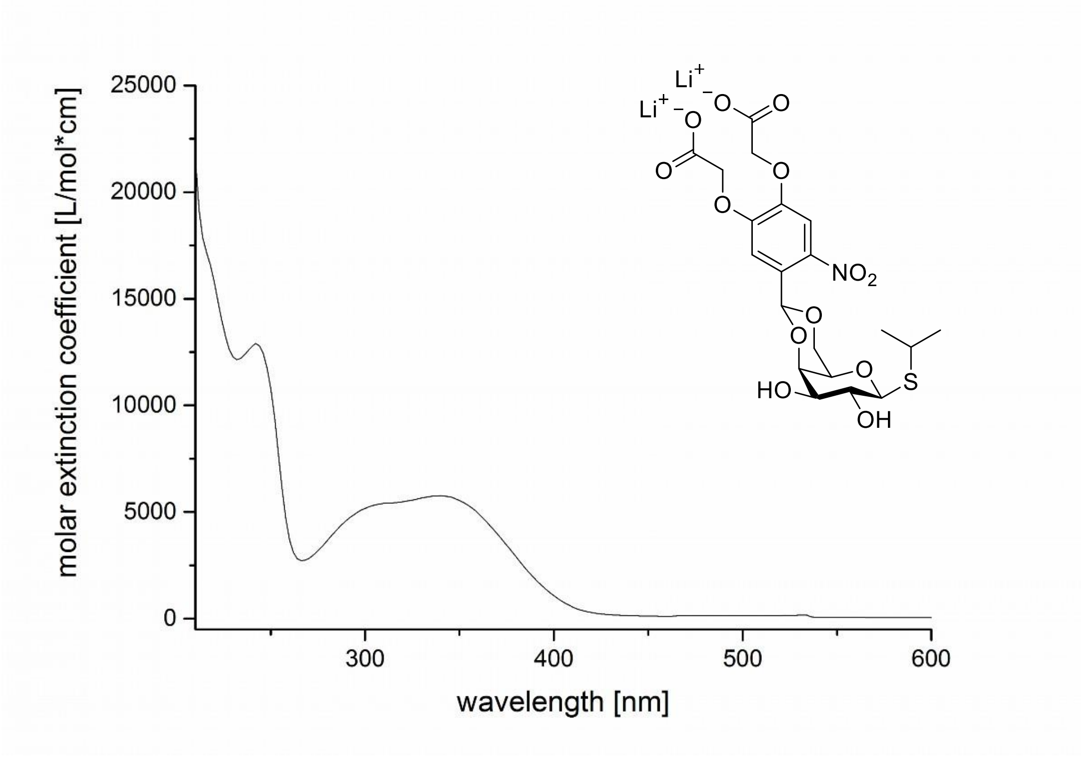

Figure S2: UV-Vis spectrum of compound **10b** [0.125 mM in sodium phosphate buffer (0.1 mM, pH 7.5), 25 °C].

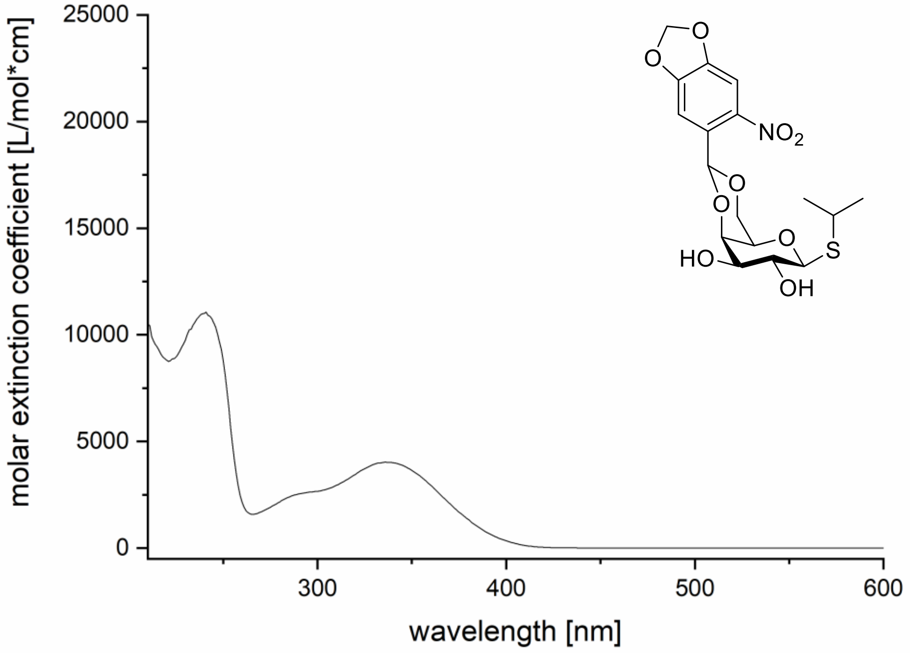

Figure S3: UV-Vis spectrum of compound **1** (0.200 mM in MeOH, 25 °C).

S2.2 Determination of uncaging half-life times

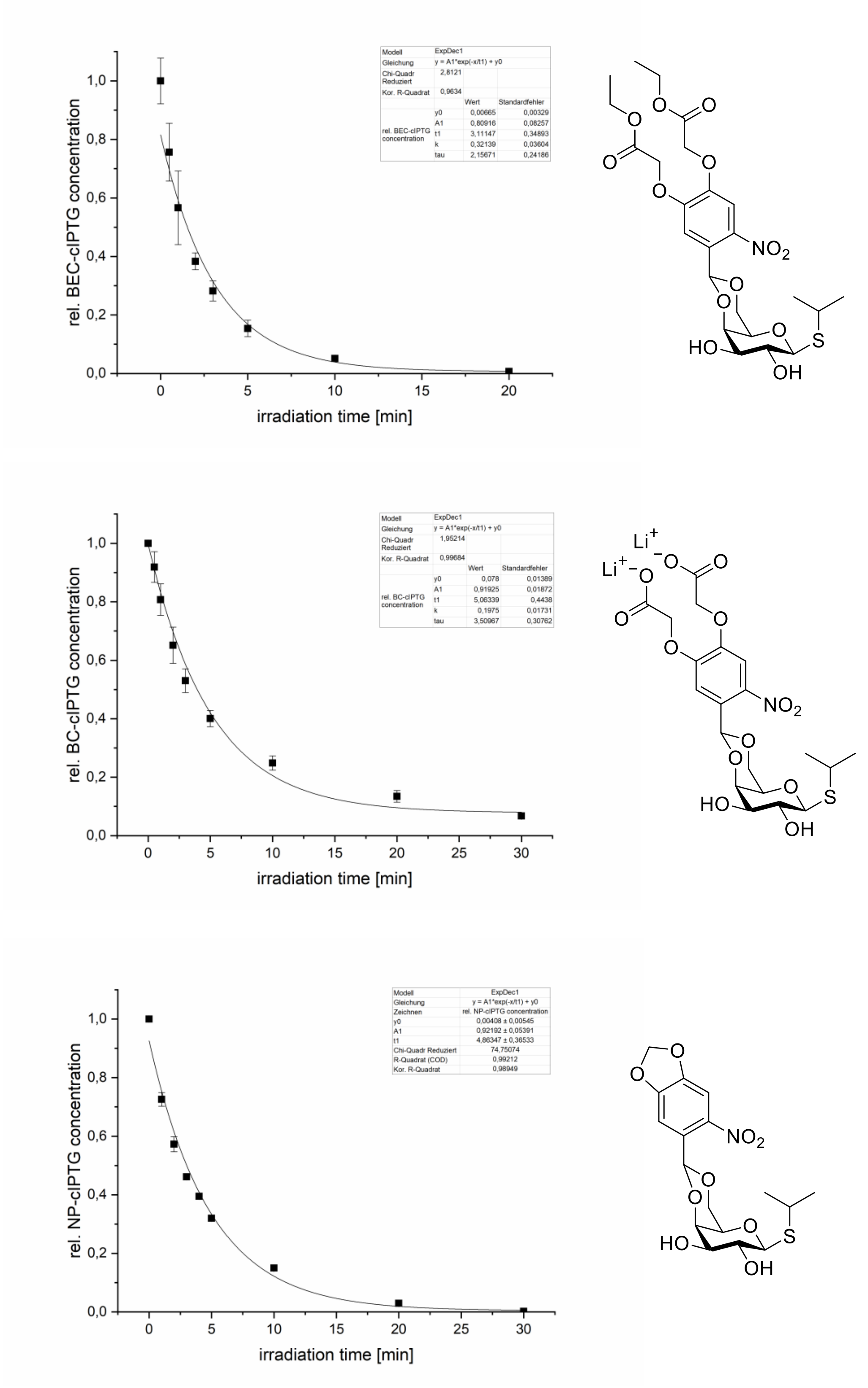

Figure S4: *In vitro* decay of caged IPTG controlled via reverse phase HPLC of compounds **10a**, **1** (1 mM in MeOH) and **10b** [1 mM in sodium phosphate buffer (0.1 mM, pH 7.5)]; 375 nm, 6.4 mW cm^{-2}, room temperature.

Table S2: Fitting parameters and uncaging half-life times $t_{0.5}$ for caged IPTG derivatives.

**Inducer**	**y_0**	**A_1**	**t_1**	**k**	**$t_{0.5}$ [min]**
BEC-cIPTG (**10a**)	0.00665	0.80916	3.11147	0.32139	2.15671
BC-cIPTG (**10b**)	0.078	0.91925	5.06339	0.1975	3.50967
NP-cIPTG (**1**)	0.00408	0.92192	4.86347	0.20561	3.3711

S2.3 Determination of purity by qNMR

Table S3: Compound purities determined by qNMR

**Compound**	**Purity [%]**
BEC-cIPTG (**10a**)	90.7 ± 1.3
BC-cIPTG (**10b**)	74.0 ± 2.5
NP-cIPTG (**1**)	80.4 ± 2.3

S2.4 Determination of IPTG concentrations sufficient for the induction of gene expression in *E. coli*, *P. putida* and *B. subtilis*

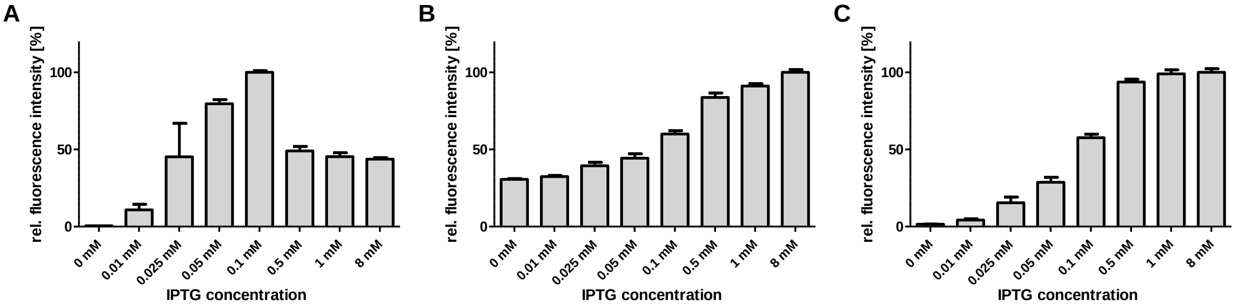

Figure S5: Relative fluorescence intensities of *E. coli* Tuner(DE3)/pRhotHi-2-lacI-eYFP (**A**), *P. putida* KT2440/pVLT33-GFPmut3 (**B**) and *B. subtilis* DB430/pHT01-sfGFP (**C**) expression cultures supplemented with increasing amounts of IPTG (**14**). Cultures were incubated in LB medium for 20 h in the dark at 30 °C. Induction of gene expression was performed after 2.5 h for *E. coli*, after 3 h for *P. putida* and after 5 h for *B. subtilis* by adding IPTG (**14**) concentrations ranging from 0 to 8 mM. *In vivo* fluorescence intensities were determined by using a BioLector system (eYFP: λex = 508 nm, λem = 532 nm, GFPmut3: λex = 508 nm, λem = 532 nm, sfGFP: λex = 488 nm, λem = 520 nm) and normalised to cell densities. Values are means of triplicate measurements. Error bars indicate the respective standard deviations.

S2.5 Stability and toxicity of novel photocaged IPTG variants

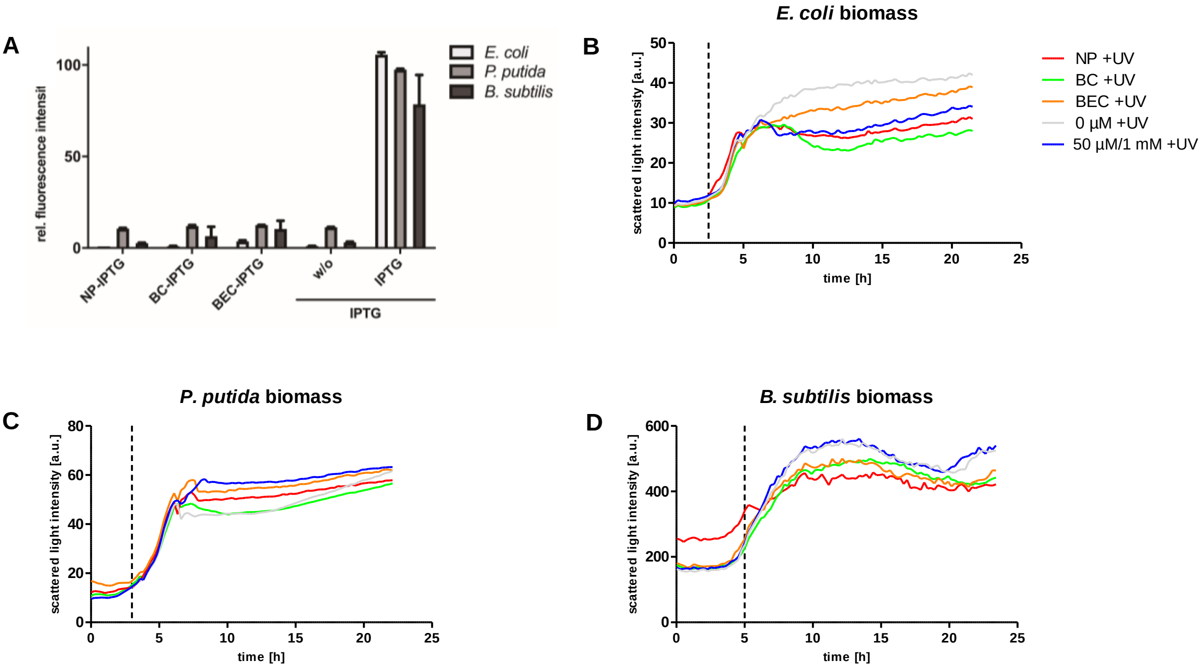

Figure S6: A) Normalised fluorescence intensity of *E. coli* Tuner(DE3)/pRhotHi-2-lacI-eYFP, *P. putida* KT2440/pVLT33-GFPmut3 and *B. subtilis* DB430/pHT01-sfGFP expression cultures (supplemented with 50 µM of each compound for *E. coli* and 1 mM for *P. putida* and *B. subtilis*). All cultures were incubated in the dark for 20 h in LB medium at 30 °C. *In vivo* stability of new photocaged IPTG variants **10a** (BEC) and **10b** (BC) is reflected by the low induction of reporter gene expression and was compared to NP-cIPTG (**1**, NP) as well as to cultures with and without IPTG (**14**). *In vivo* fluorescence intensities were determined by using a BioLector system (eYFP: λex = 508 nm, λem = 532 nm, GFPmut3: λex = 508 nm, λem = 532 nm, sfGFP: λex = 488 nm, λem = 520 nm) normalised to cell densities and are shown in relation to the respective fluorescence intensities of IPTG (**14**). Values are means of triplicate measurements. Error bars indicate the respective standard deviations. **B-D)** Growth curves of *E. coli* Tuner(DE3)/pRhotHi-2-lacI-eYFP, *P. putida* KT2440/pVLT33-GFPmut3 and *B. subtilis* DB430/pHT01-sfGFP expression cultures in the presence of novel photocaged IPTG variants **10a** (BEC) and **10b** (BC) compared to **1** (NP) as well as uninduced (0 mM) and induced (50 µM/1 mM of **14**). Cells were grown over 20 h using a BioLector system (LB medium supplemented with 50 µM of each caged compound for *E. coli* and 1 mM for *P. putida* and *B. subtilis*, 30 °C, 1200 rpm). Cell growth was analysed by determining the scattered light intensity. After 2.5, 3, and 5 h, formation of photoproducts was induced in cultures of *E. coli*, *P. putida* and *B. subtilis via* light exposure at 365 nm (~1 mW cm^{-2}, indicated by dashed lines) for 30 min or by the addition of conventional IPTG (**14**). Values are means of triplicate measurements.

S2.6 Effect of UV-A light illumination on cell growth and fluorescence of *E. coli*, *P. putida* and *B. subtilis* expression cultures

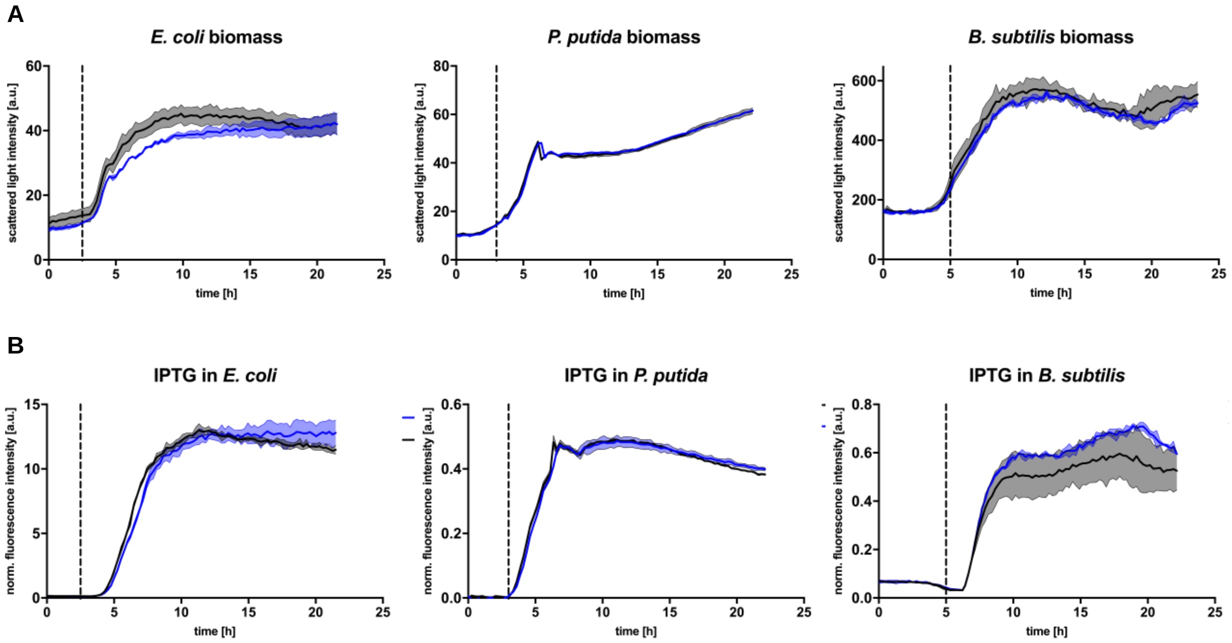

Figure S7: A) Growth curves of *E. coli* Tuner(DE3)/pRhotHi-2-lacI-eYFP, *P. putida* KT2440/pVLT33-GFPmut3 and *B. subtilis* DB430/pHT01-sfGFP expression cultures in the presence (blue line) and absence (black line) of UV-A light. Cells were grown over 20 h using a BioLector system (LB medium without inducer, 30 °C, 1200 rpm). Cell growth was analyzed by determining the scattered light intensity. After 2.5h (*E. coli*), 3 (*P. putida*), and 5 h (*B. subtilis*), cultures were exposed to UV-A light at 365 nm (~1 mW cm^{-2}, indicated by dashed lines) for 30 min. Values are means of triplicate measurements. Error bars indicate the respective standard deviations. **B)** Increase of fluorescent reporter-mediated signals during cultivation of *E. coli* Tuner(DE3)/pRhotHi-2-lacI-eYFP, *P. putida* KT2440/pVLT33-GFPmut3 and *B. subtilis* DB430/pHT01-sfGFP expression cultures with (blue line) and without (black line) UV-A exposure at 365 nm (~1 mW cm^{-2}) for 30 min; cells were grown over 20 h in LB medium at 30 °C and 1200 rpm using a BioLector system. Induction was performed using 50 µM of IPTG (**7**) for *E. coli* and 1 mM IPTG for *P. putida* and *B. subtilis*. The individual time point of induction is indicated by the dashed lines (*E. coli* 2.5 h, *P. putida* 3 h, *B. subtilis* 5 h). *In vivo* fluorescence intensities were determined by using a BioLector system (eYFP: λex = 508 nm, λem = 532 nm, GFPmut3: λex = 508 nm, λem = 532 nm, sfGFP: λex = 488 nm, λem = 520 nm) and normalised to cell densities. Values are means of triplicate measurements.

S2.7 Comparative analysis of light-responsiveness of cIPTG variants in different bacterial expression systems

In order to analyse the light-responsiveness of BC-, BEC-, and NP-cIPTG in different bacterial expression hosts, fluorescent protein expression was online monitored during batch cultivation in LB medium at 30 °C and 1200 rpm using a BioLector system (eYFP: λex = 508 nm, λem = 532 nm, GFPmut3: λex = 508 nm, λem = 532 nm, sfGFP: λex = 488 nm, λem = 520 nm). To analyse time-resolved fluorescent protein signals of *E. coli* Tuner(DE3)/pRhotHi-2-lacI-eYFP, *P. putida* KT2440/pVLT33-GFPmut3 and *B. subtilis* DB430/pHT01-sfGFP expression strains, cultures were supplemented with IPTG (**7**), BEC-cIPTG (**10a**), BC-cIPTG (**10b**), and NP-cIPTG (**1**). Induction of gene expression was performed after 2.5 h, 3 h or 5 h for *E. coli*, *P. putida* and *B. subtilis*, respectively, *via* UV-A light exposure at 365 nm (~1 mW cm^{-2}) for 30 min or the addition of **14**. Cell density-normalised fluorescence signal curves (Fig. S8) were plotted and fitted to a sigmoidal Boltzmann fit using GraphPad Prism 5.03 ®. The half-maximal responsiveness of each cIPTG variant was calculated from fitting parameters (Table S4) using the following standard equation for sigmoidal Boltzmann fitting:

$$y = \frac{A_1 - A_2}{1 + e^{(x - x_{0)idx}}} + A_2$$

Table S4: Calculation of half-maximal responsiveness $t_{0.5}$ for the lacI/P_{tac}/P_{T7}/P_{grac}-regulated systems using fitting parameters from sigmoidal Boltzmann fits. Final half-maximal responsiveness $t_{0.5}$ final was calculated as the difference of $t_{0.5}$ calc. and the induction time point t_0. ($y_{0.5}$ = fluorescence intensity at half-maximal time value, A1 = initial value, A2 = final value, x_0 = center value, dx = time constant)

**Inducer**	**$y_{0.5}$**	**A1**	**A2**	**x_0**	**dx**	**$t_{0.5}$ [h] calc.**	**t_0 [h]**	**$t_{0.5}$ [h] final**
***E. coli* Tuner(DE3)/pRhotHi-2-lacI-eYFP**								
**NP-IPTG (1)**	6.36	-0.12	12.36	6.87	1.21	6.91	2.5	4.41
**BC-IPTG (10b)**	5.52	-0.16	10.55	6.59	1.41	6.66	2.5	4.16
**BEC-IPTG (10a)**	4.36	-0.01	8.68	7.01	0.91	7.01	2.5	4.51
**IPTG (14)**	6.49	-0.13	12.61	6.71	0.99	6.75	3.0	3.75
***P. putida* KT2440/pVLT33-GFPmut3**								
**NP-IPTG (1)**	0.06	-0.01	0.10	6.37	0.78	6.58	3.0	3.58
**BC-IPTG (10b)**	0.15	-0.01	0.28	5.55	0.67	5.62	3.0	2.62
**BEC-IPTG (10a)**	0.09	0.00	0.17	5.87	0.82	5.94	3.0	2.94
**IPTG (14)**	0.23	0.00	0.45	4.89	0.61	4.91	3.5	1.41
***B. subtilis* DB430/pHT01-sfGFP**								
**NP-IPTG (1)**	0.11	0.04	0.34	7.25	0.60	6.96	5.0	1.96
**BC-IPTG (10b)**	0.25	0.06	0.68	7.52	0.61	7.31	5.0	2.31
**BEC-IPTG (10a)**	0.17	0.06	0.51	7.30	0.68	6.97	5.0	1.97
**IPTG (14)**	0.24	0.05	0.64	7.80	0.68	7.57	5.5	2.07

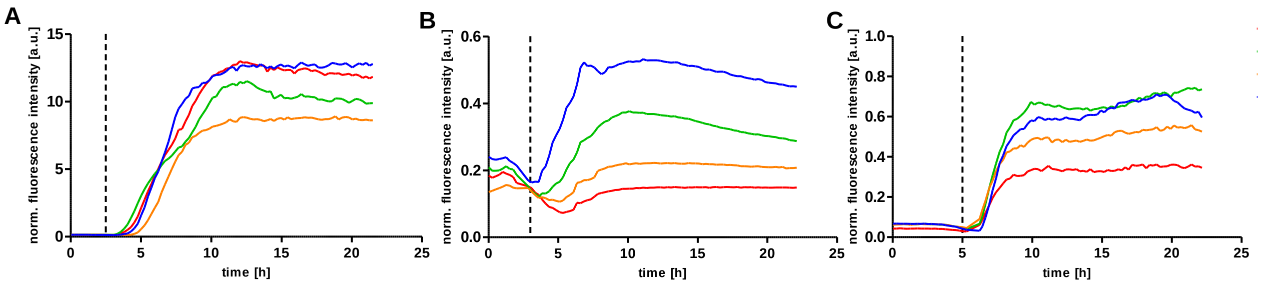

Figure S8: Normalised fluorescence protein expression profiles of *E. coli* Tuner(DE3)/pRhotHi-2-lacI-eYFP (**A**), *P. putida* KT2440/pVLT33-GFPmut3 (**B**) and *B. subtilis* DB430/pHT01-sfGFP (**C**) cultures supplemented with IPTG (**7**; blue line), BEC-cIPTG (**10a**; orange line), BC-cIPTG (**10b**; green line), and NP-cIPTG (**1**; red line) (50 µM of each compound were used for *E. coli* and 1 mM for *P. putida* and *B. subtilis*). Cells were grown over 20 h in LB medium at 30 °C and 1200 rpm using a BioLector system. Induction was performed using UV-A exposure at 365 nm (~1 mW cm^{-2}) for 30 min or respective amount of IPTG (**14**). Time of induction is indicated by dashed lines (*E. coli* 2.5 h, *P. putida* 3 h, *B. subtilis* 5 h). *In vivo* fluorescence intensities were online-monitored during cultivation (eYFP: λex = 508 nm, λem = 532 nm, GFPmut3: λex = 508 nm, λem = 532 nm, sfGFP: λex = 488 nm, λem = 520 nm) and normalised to cell densities. Values are means of triplicate measurements.

Table S5: Calculation of induction factors for IPTG and light-responsive cIPTG in *E. coli* Tuner(DE3)/pRhotHi-2-lacI-eYFP, *P. putida* KT2440/pVLT33-GFPmut3 and *B. subtilis* DB430/pHT01-sfGFP. Cultures were supplemented with IPTG (**14**), BEC-cIPTG (**10a**), BC-cIPTG (**10b**), and NP-cIPTG (**1**) in concentrations of 50 µM of each compound for *E. coli* and 1 mM for *P. putida* and *B. subtilis*. These values correspond to Figure 2 shown in the result section.

**Inducer**	**Induction factor**		
	E. coli	***P. putida***	***B. subtilis***
NP-cIPTG **(1)**	114.93±3.47	3.03±0.10	20.14±1.22
BC-cIPTG **(10b)**	87.57±21.15	5.47±0.12	21.80±11.94
BEC-cIPTG **(10a)**	23.45±2.36	3.62±0.20	8.20±2.67
IPTG (-UV-A)	96.80±2.56	8.65±0.14	25.62±2.21

S2.8 Heterogeneity of light-induced reporter gene expression in *E. coli* and *B. subtilis* using different cIPTG variants

Caged inducer variants **10a** (BEC), **10b** (BC) and **1** (NP) were used in comparison to conventional IPTG (**14**) to analyse the heterogeneity of reporter gene expression in *E. coli* Tuner(DE3)/pRhotHi-2-lacI-eYFP (**A, C**) and *B. subtilis* DB430/pHT01-sfGFP (**B, D**) cultures. To this end, fluorescence intensity and fluorescence distribution of 10,000 cells of a population were determined using flow cytometry when cultures reached the late logarithmic growth phase (8 h for *E. coli* and 10 h for *B. subtilis*).

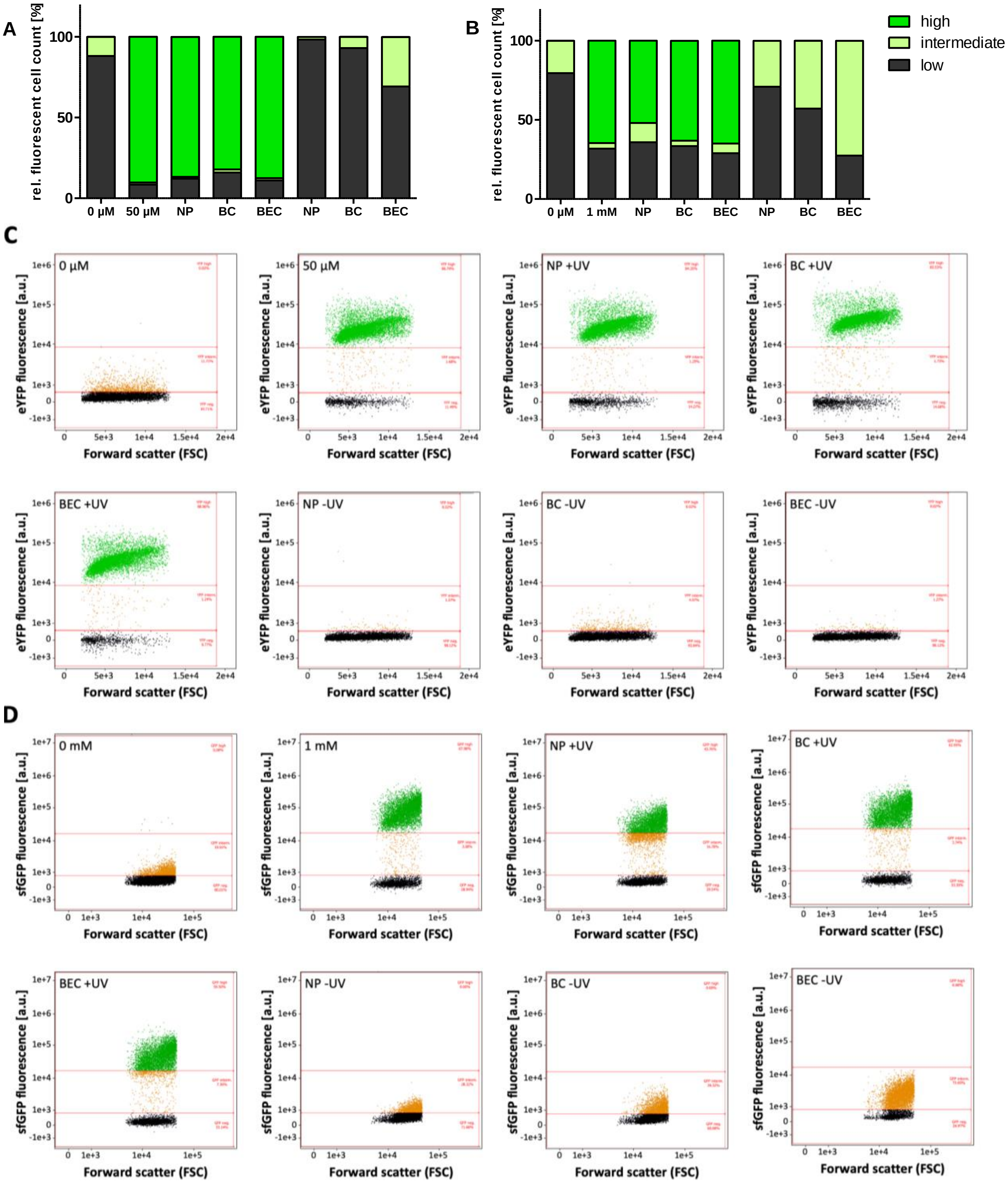

Figure S9: Single cell analysis of *E. coli* Tuner(DE3)/pRhotHi-2-lacI-eYFP (**A**) and *B. subtilis* DB430/pHT01-sfGFP (**B**) expression cultures supplemented with 50 µM of each caged compound for *E. coli* and 1 mM for *B. subtilis*. Induction was

performed after 2.5 h for *E. coli* and after 5 h for *B. subtilis* using UV-A light (30 min, ~1 mW cm^{-2}) or common IPTG (**14**). As a negative control, cells of both species were identically cultivated but kept in the dark. Culture samples were collected after late logarithmic growth phase (8 h for *E. coli* and 10 h for *B. subtilis*) and analysed using flow cytometry. The cells were gated based on FSC and SSC to exclude cell debris and accumulation of cells. The fluorescence intensities of eYFP or sfGFP were measured using a 488 nm-laser and a 528/46 nm bandpass filter and intensity values were classified into the three categories "high", "intermediate" and "low". All values are means of triplicate measurements. Raw data of the diagrams depicted in (**A**) and (**B**) are shown for *E. coli* Tuner(DE3)/pRhotHi-2-lacI-eYFP (**C**) and *B. subtilis* DB430/pHT01-sfGFP (**D**). Initially, the cells were gated based on their respective FSC and SSC signals to exclude cell debris and accumulation of cells. Afterwards, they were analysed regarding both their eYFP or sfGFP fluorescence intensity and their forward scatter signal (FSC). All graphs are representative examples of triplicate measurements.

S2.9 NMR spectra of compounds

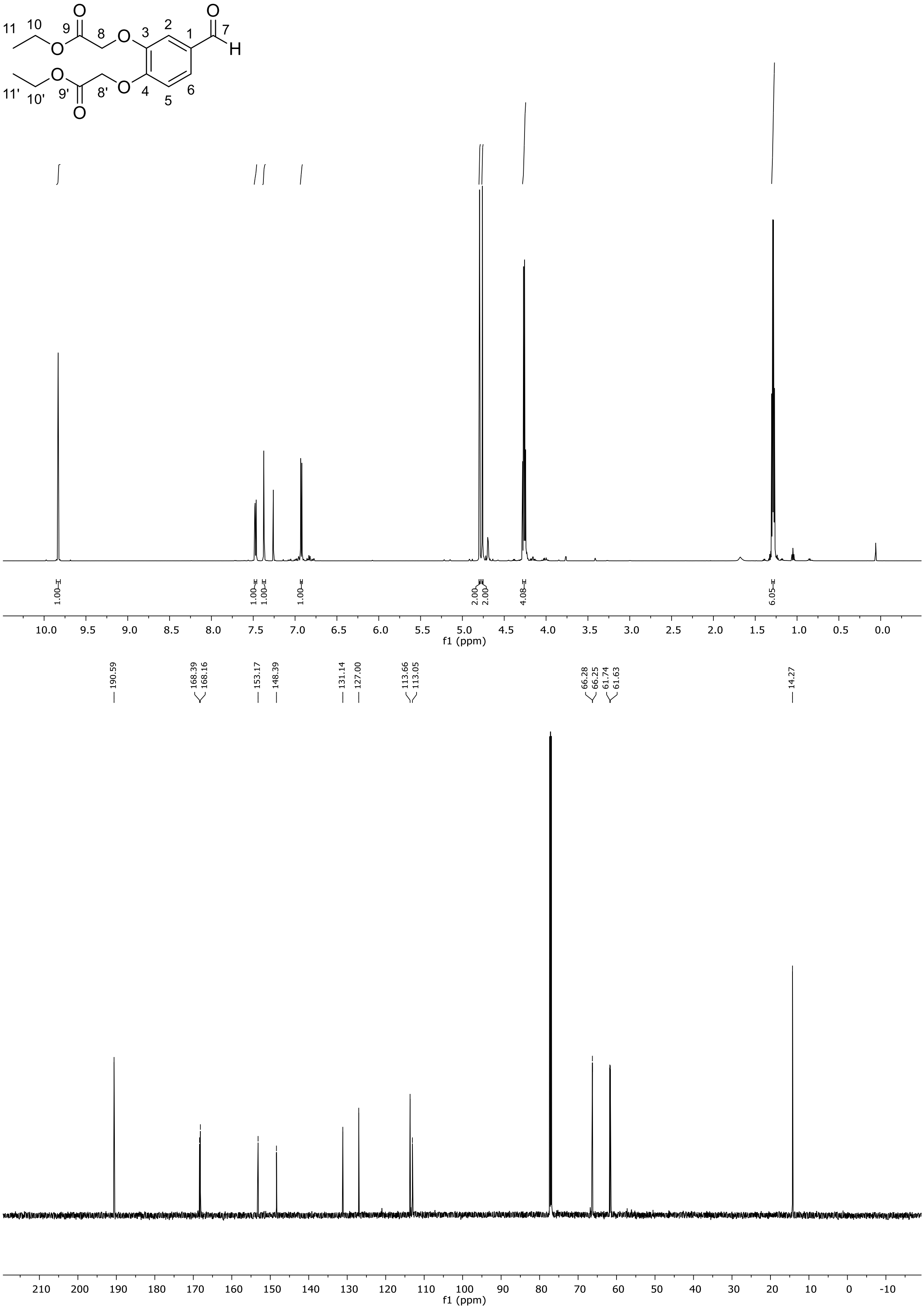

Figure S10: ^{1}H- and ^{13}C-NMR spectra of **18** in $CDCl_3$ (600 MHz/151 MHz).

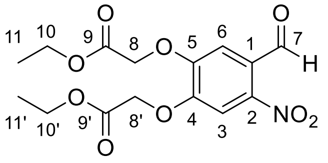

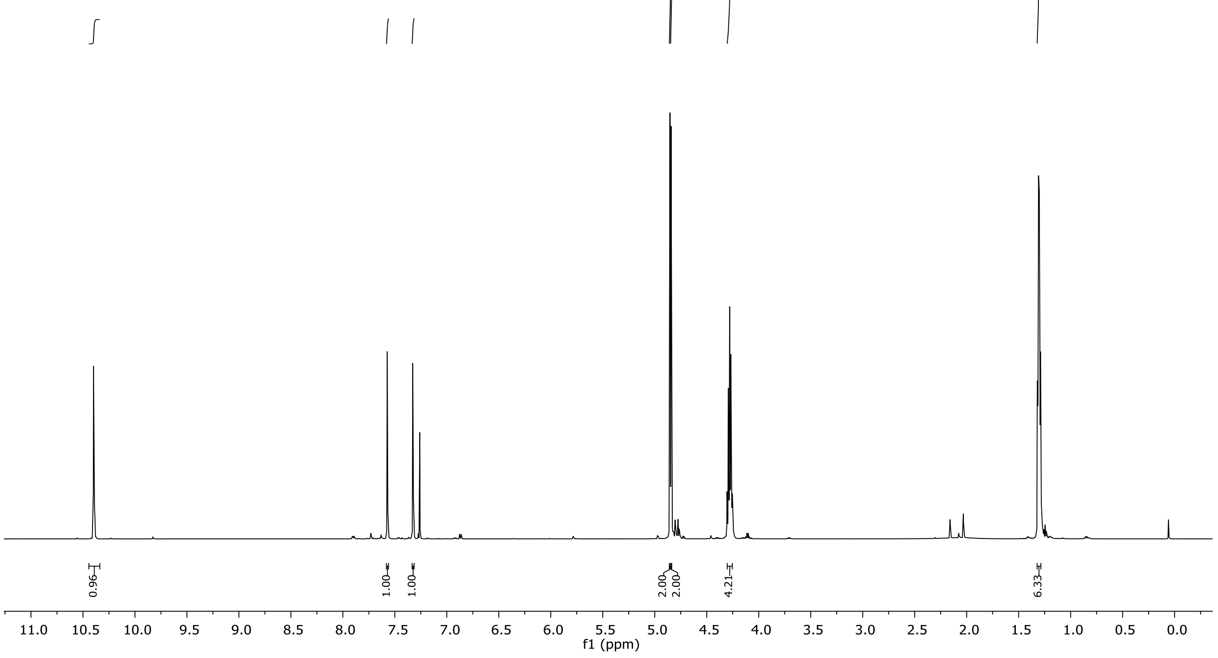

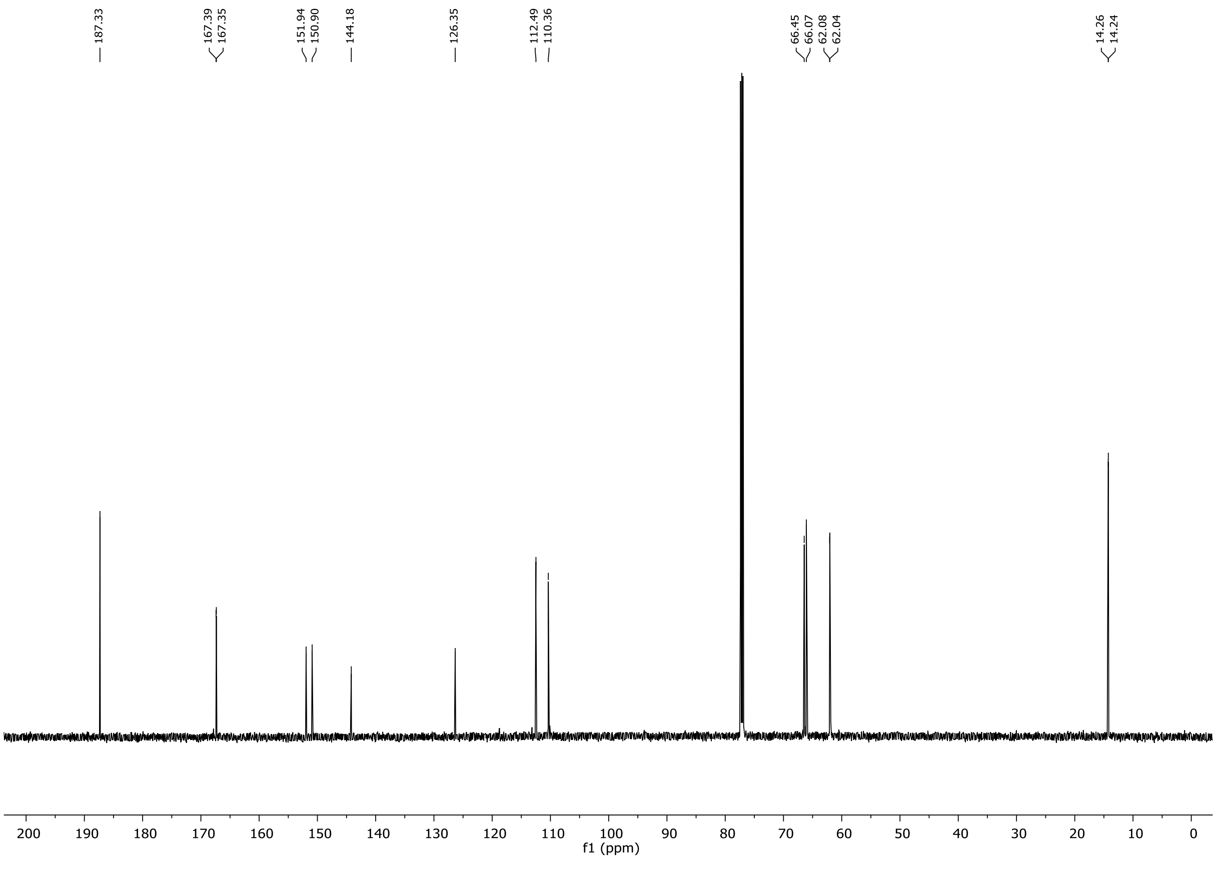

Figure S11: ^{1}H- and ^{13}C-NMR spectra of **11** in $CDCl_3$ (600 MHz/151 MHz).

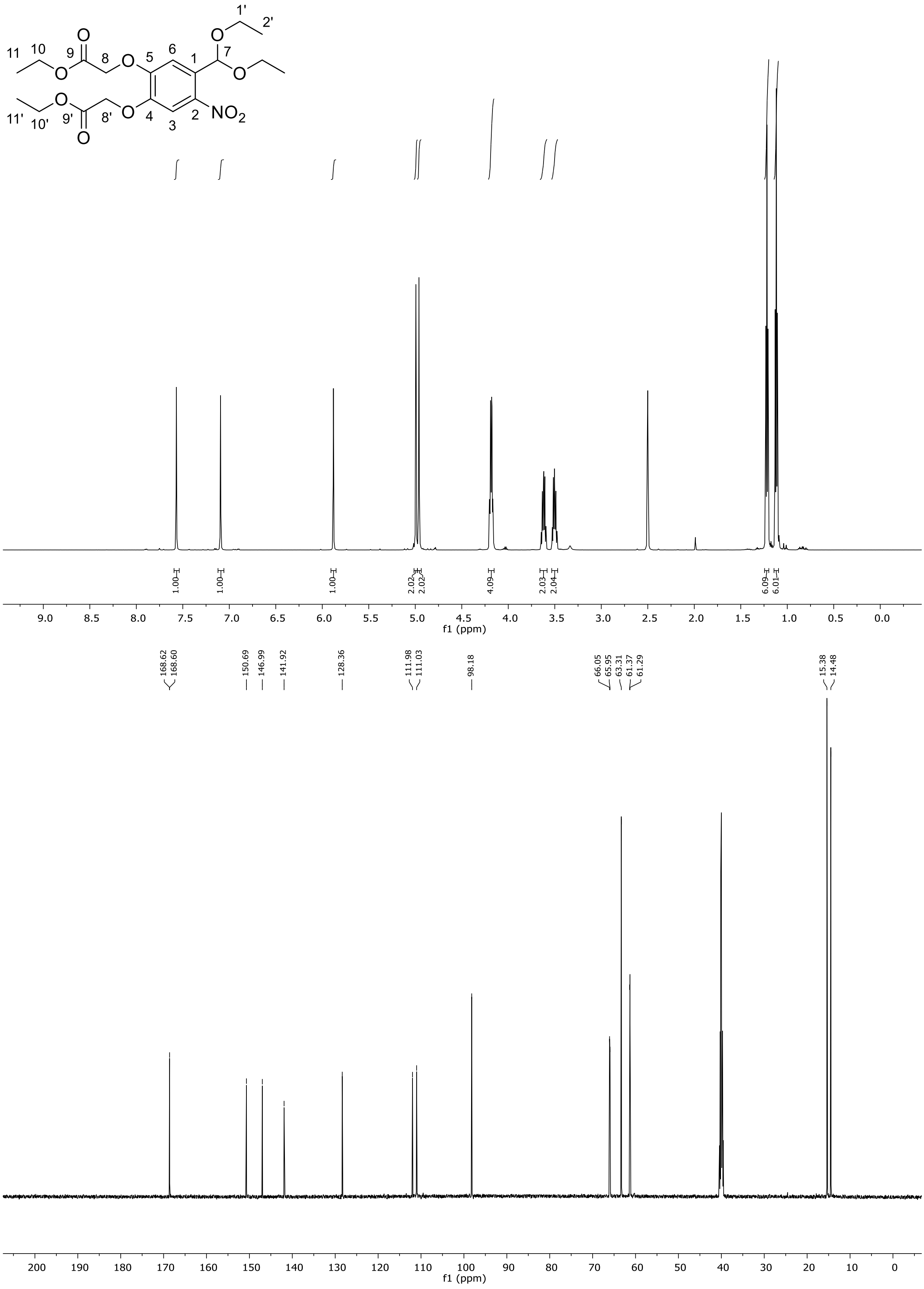

Figure S12: ^{1}H- and ^{13}C-NMR spectra of **12** in DMSO (600 MHz/151 MHz).

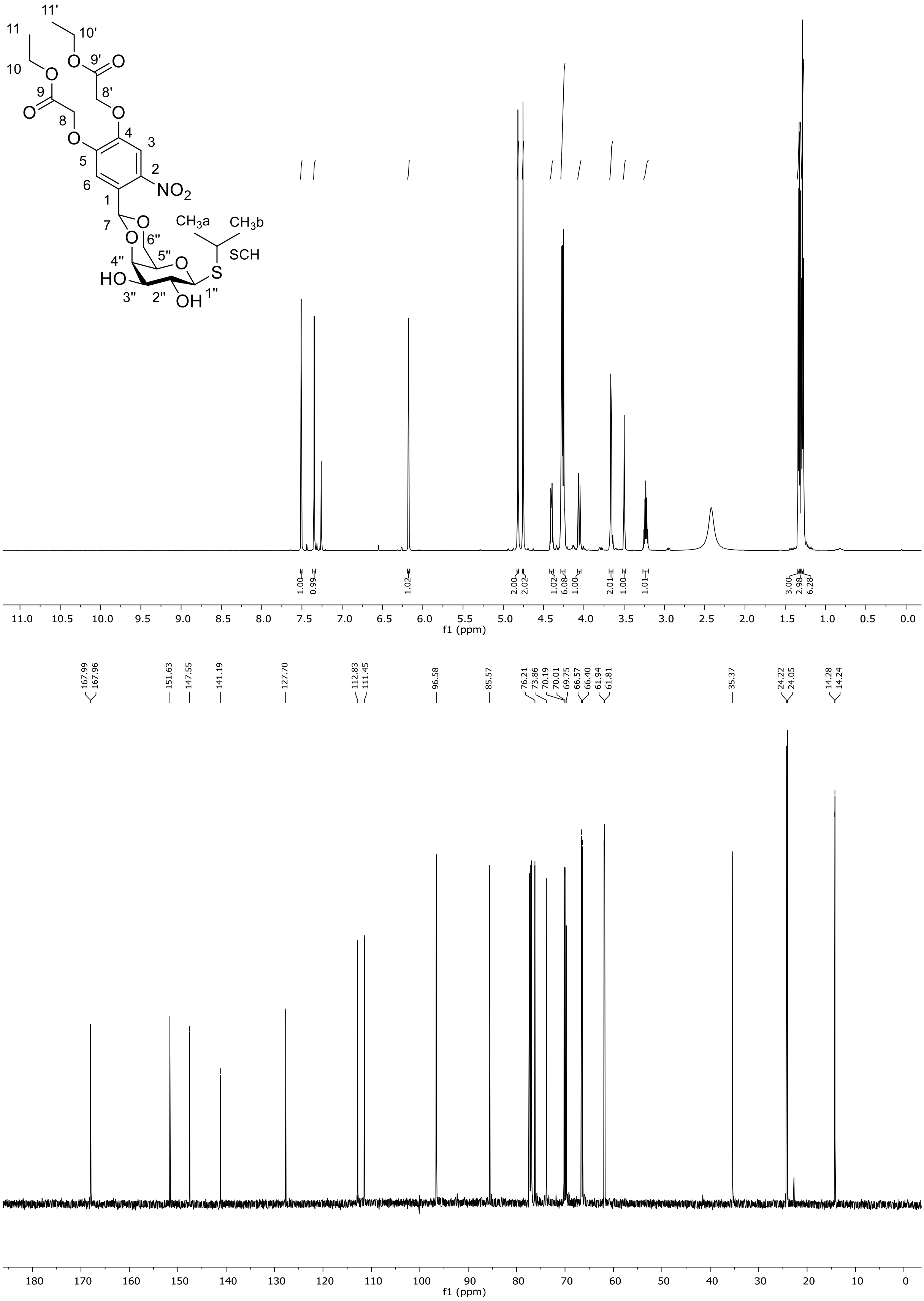

Figure S13: ^{1}H- and ^{13}C-NMR spectra of **10a** in $CDCl_3$ (600 MHz/151 MHz).

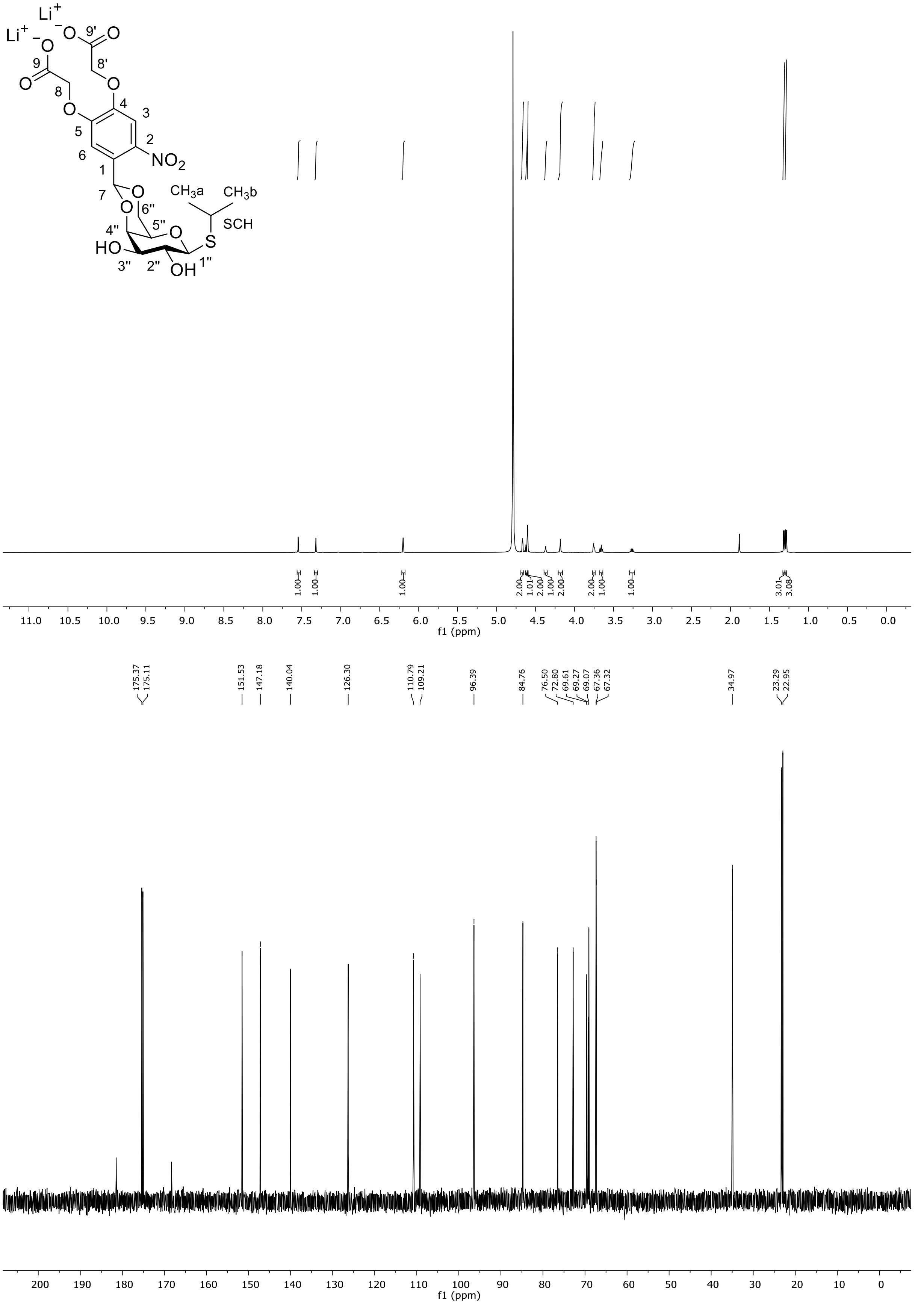

Figure S14: ^{1}H- and ^{13}C-NMR spectra of **10b** in D_2O (600 MHz/151 MHz).

S2.10 HPLC-Traces

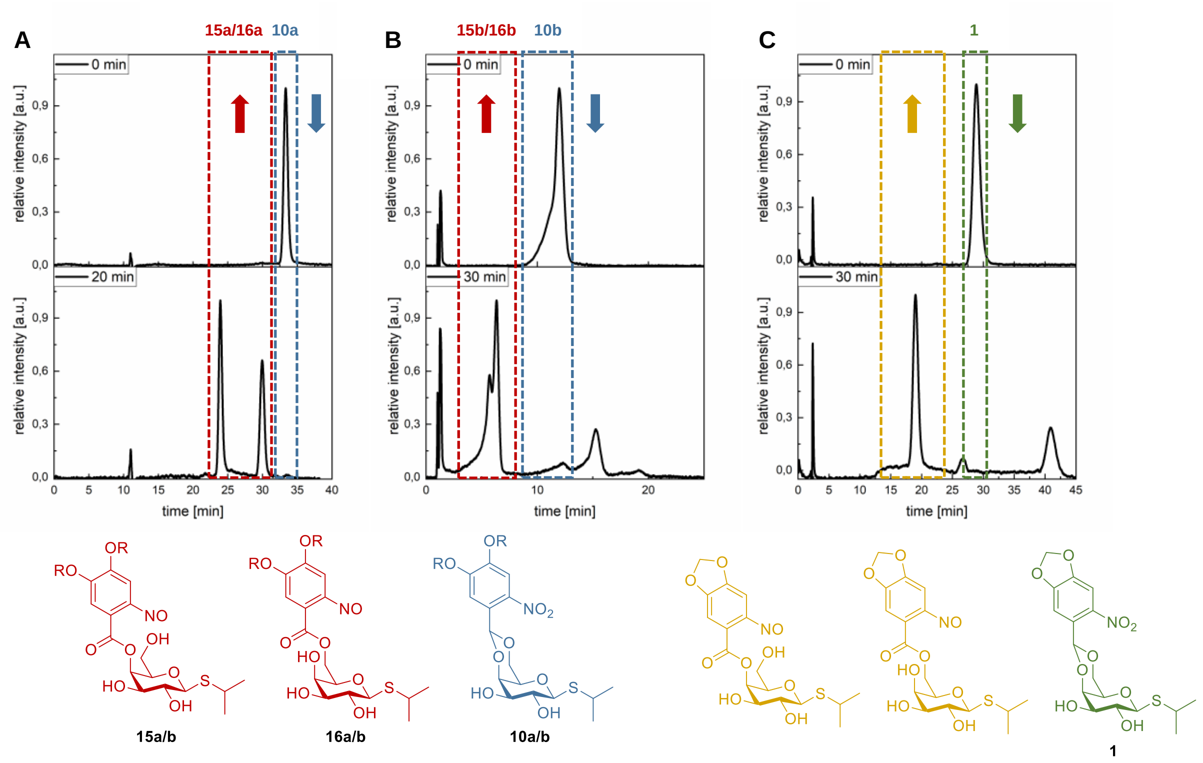

10a, **15a**, **16a**: R = CH_2CO_2Et
10b, **15b**, **16b**: R = $CH_2CO_2^-$ Li^+

Figure S15: A) UV trace at 298 nm of the reverse phase HPLC analysis of BEC-cIPTG (**10a**) (1 mM in MeOH) before irradiation and after 20 min of irradiation (375 nm, 6.4 mW cm^{-2}, room temperature). **B**) UV trace at 340 nm of the reverse phase HPLC analysis of BC-cIPTG (**10b**) [1 mM in sodium phosphate buffer (0.1 mM, pH 7.5)] before irradiation and after 30 min of irradiation (375 nm, 6.4 mW cm^{-2}, room temperature). **C**) UV trace at 336 nm of the reverse phase HPLC analysis of NP-cIPTG (**1**) (1 mM in MeOH) before irradiation and after 30 min of irradiation (375 nm, 6.4 mW cm^{-2}, room temperature). Assignment of photoproducts for **A**), **B**) and **C**) was performed by observation of shifts in the UV-spectrum, which were in accordance to previously investigated derivatives.[9]

[1] J. Ni, D. A. Auston, D. A. Freilich, S. Muralidharan, E. A. Sobie, J. P. Y. Kao, *J. Am. Chem. Soc.* **2007**, *129*, 5316-5317.

[2] E. Brunet, M. a. T. Alonso, O. Juanes, O. Velasco, J. C. Rodríguez-Ubis, *Tetrahedron* **2001**, *57*, 3105-3116.

[3] D. Hanahan, *J. Mol. Biol.* **1983**, *166*, 557-580.

[4] R. Simon, U. Priefer, A. Pühler, *Bio/Technology* **1983**, *1*, 784-791.

[5] M. Bagdasarian, R. Lurz, B. Rückert, F. C. H. Franklin, M. M. Bagdasarian, J. Frey, K. N. Timmis, *Gene* **1981**, *16*, 237-247.

[6] R. H. Doi, S.-L. Wong, F. Kawamura, *Trends Biotechnol.* **1986**, *4*, 232-235.

[7] D. Binder, A. Grunberger, A. Loeschcke, C. Probst, C. Bier, J. Pietruszka, W. Wiechert, D. Kohlheyer, K.-E. Jaeger, T. Drepper, *Integr. Biol.* **2014**, *6*, 755-765.

[8] V. de Lorenzo, L. Eltis, B. Kessler, K. N. Timmis, *Gene* **1993**, *123*, 17-24.

[9] a) F. Bley, K. Schaper, H. Görner, *Photochem. Photobiol.* **2008**, *84*, 162-171; b) C. Bier, Heinrich Heine University Düsseldorf (Düsseldorf, Germany), **2017**.
